# The role of ovarian hormones in risk aversion in female rats

**DOI:** 10.1038/s41386-026-02347-9

**Published:** 2026-01-26

**Authors:** Leah M. Truckenbrod, Nadia Carlos, Megan Kelly, Merrick Garner, Madeline Streifer, Andrea C. Gore, Caitlin A. Orsini

**Affiliations:** 1 Department of Neuroscience, University of Texas at Austin, Austin, TX USA; 2 Department of Psychology, University of Texas at Austin, Austin, TX USA; 3 Division of Pharmacology and Toxicology, University of Texas at Austin, Austin, TX USA; 4 Department of Neurology, University of Texas at Austin, Austin, TX USA; 5 Waggoner Center for Alcohol and Addiction Research, University of Texas at Austin, Austin, TX USA

**Keywords:** Motivation, Reward

## Abstract

Decision making involving risk of punishment is a cognitive process characterized by sex-specific phenotypes, with females exhibiting greater risk aversion than males. Although prior research has demonstrated that ovarian hormones, and estradiol (E2) in particular, contribute to increased risk aversion in females, the receptor mechanisms underlying these effects remain unknown. Further, it is unclear what role the other key ovarian hormone progesterone (P4) plays in female risk aversion. Accordingly, the current set of experiments were designed to address these gaps in knowledge of the hormonal basis of female risk-taking behavior. Female rats were trained in a punishment-based risky decision-making task, ovariectomized, and then retested in the decision-making task. Rats were then treated with estradiol benzoate (Experiment 1; EB), estrogen receptor (ER) agonists (Experiment 2) or progesterone (Experiment 3; P4) after daily test sessions for 7 days. Consistent with prior work, OVX increased risk taking, and EB administration attenuated this effect. Administration of an ERα agonist, either alone or with an ERβ agonist, similarly mitigated the effects of OVX on risk taking. In contrast, the ERβ agonist alone was ineffective in restoring risk aversion in OVX females. Control tests confirmed that the effects of the ERα agonist on risk taking were not due to altered food motivation or footshock sensitivity. Finally, P4 administration did not alter risk taking in OVX females and did not inhibit EB’s behavioral effects. Collectively, these data reveal that E2 is the critical ovarian hormone that promotes female risk aversion; further, they suggest that the likely mechanism by which E2 influences risk aversion in females is through activation of ERα.

## Introduction

There are well-established sex differences in risk-based decision making [1–6]. Although the nature of these sex differences vary depending on the cost associated with the risky option [1, 7–12], previous studies in rodents have demonstrated that when the reward is associated with potential punishment, female rats exhibit greater aversion to risky options than male rats [1, 6, 13]. Although physical punishment is rarely used in human laboratory risk-taking tasks, such sex differences in punishment-based decision making are largely congruent with those observed across multiple decision-making domains in humans, including financial risk taking, sensation-seeking and everyday risk-based choices (e.g., driving practices) [2, 14–20]. Increased aversion to risk of punishment in females (hereafter referred to as risk aversion) is mediated by gonadal hormones, as ovariectomies (OVX) increase preference for rewards associated with potential punishment (e.g., risk taking) and exogenous administration of estradiol benzoate (EB) attenuates this effect [21]. Administering EB to male rats also decreases risk taking, irrespective of their gonadal status [21]. Collectively, these findings indicate estradiol (E2) is broadly involved in biasing choice away from options associated with potential punishment. The mechanisms by which E2 mediates female risk aversion to options associated with potential punishment, however, are unknown.

E2 likely regulates risk aversion via activation of estrogen receptor α (ERα) and/or estrogen receptor β (ERβ), both of which are expressed in brain regions involved in decision making [22–24]. Unlike membrane-bound ERs, nuclear ERα and ERβ exert long-lasting behavioral effects by influencing gene transcription [25–27]. Previous work suggests decision making in females relies on the genomic action of ERs. In an effort-based decision-making task, concurrent administration of ERα and ERβ agonists decreased preference for larger, more costly (i.e., requiring greater effort) rewards in OVX rats, mimicking effects of EB administration [28]. This effect was not observed when each agonist was administered alone and only appeared 24 h after administration. These findings suggest E2-mediated effort-based decision making requires concurrent ERα and ERβ activation, and that the behavioral consequences of their activation are genomic in nature. More recently, we showed that an ERβ antagonist increases choice of large rewards associated with risk of punishment in intact females [22], implicating ERβ in E2-mediated risk aversion. It is unknown, however, whether ERα is similarly involved in female risk aversion. Further, because prior work used intact rather than OVX females [22], it is unclear whether activation of either or both ERs is sufficient to mimic effects of E2 during conditions in which levels of circulating ovarian hormones are reduced.

Ovariectomies also remove most progesterone (P4) from circulation [29]. Changes in risk taking following OVX may therefore also be due to the relative absence of P4. Indeed, P4 is necessary for certain forms of decision making. For instance, P4 decreases impulsive action and impulsive choice for cocaine in females [30, 31]. Notably, for drug-related behavior, P4 either has no effect or induces effects opposite to those produced by E2 when administered alone [32–35]; when given to OVX females concurrently with E2, P4 antagonizes E2’s behavioral effects [36–38]. Relevant to decision making involving choice between risky and safe options, P4 increases choice of food reinforcers associated with footshock punishment in a conflict task in OVX rats [39]. Hence, in contrast to E2, P4 may promote riskier choices and actually inhibit E2’s ability to reverse OVX-induced behavioral changes.

The goals of the current study were to determine the roles of ERα and ERβ in female risk aversion and identify the influence of P4 on decision making involving risk of punishment in females. We hypothesized that female risk aversion requires the activation of ERβ and that P4 biases choice toward riskier options and counteracts E2’s ability to reduce risk taking. Collectively, this work provides novel information about the hormonal processes that contribute to decision making involving risk of explicit punishment in females.

## Materials and methods

### Subjects

Female Long-Evans rats (n = 100) were singly housed and maintained on a reverse light/dark cycle. During testing, rats had free access to water but were food-restricted to 85% of their free-feeding weight. All procedures were approved by The University of Texas at Austin Institutional Animal Care and Use Committee and adhered to guidelines of the National Institutes of Health. Additional information about subjects and housing conditions are provided in the Supplementary Material.

### Overview of experimental design

**Fig. 1:**
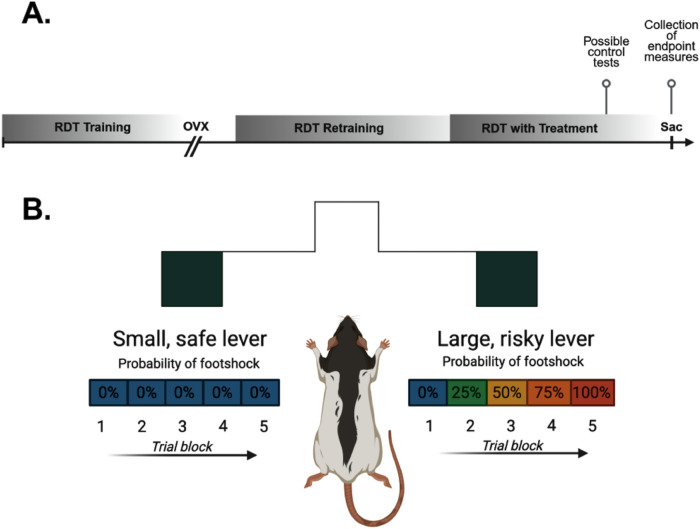
Overview of experimental timeline and schematic of the Risky Decision-making Task (RDT). **A** General experimental timeline for all experiments. **B** In the RDT, rats are given a choice between two levers in an operant chamber. A press on one lever results in the delivery of 1 food pellet (small, safe lever), whereas a press on the other lever results in the delivery of a larger food reward accompanied by an increasing probability of footshock delivery (large, risky lever). Illustrations generated with Biorender.com.

In all experiments, rats were tested in the Risky Decision-making Task (RDT) until they reached behavioral stability, underwent OVX and then re-tested in the RDT until stability re-emerged (Fig. 1A).

The goal of Experiment 1 (n = 20) was to confirm effects of OVX on risk taking and the ability of EB to attenuate these effects. After reaching stability post-OVX, rats were treated with EB or vehicle 1–2 h after daily testing.

Experiment 2 (n = 40) determined the roles of ERα and ERβ in female risk aversion. After reaching stability post-OVX, rats (n = 20) received injections of an ERα agonist, an ERβ agonist, both agonists together or vehicle 1–2 h after daily test sessions. Following RDT testing, rats were tested on a Progressive Ratio (PR) schedule of reinforcement and shock reactivity (SR) assay under the different ER agonist conditions using a between-subjects design. To increase the sample size of the groups in these assays, a separate cohort of rats (n = 20) underwent OVX and were tested on the PR and SR assays under the different ER agonist treatments. These rats were then treated with the ERβ agonist or vehicle and tested in an open field assay to provide a positive behavioral control for the ERβ agonist dose.

Experiment 3 (n = 20) determined the effects of P4 on risk taking in OVX females with or without EB. After reaching stability post-OVX, rats received P4 alone, P4 and EB together or vehicle 1–2 h after daily test sessions.

### Surgery

A detailed description of surgical procedures is provided in the Supplementary Material. Briefly, rats were anesthetized under isoflurane and underwent OVX. After one week of recovery, rats were food-restricted in preparation for resuming behavioral testing in the RDT.

### Behavioral procedures

A comprehensive description of all behavioral equipment and initial behavioral training procedures are provided in the Supplementary Material.

#### Risky decision-making task (RDT)

Behavioral training occurred in ten operant chambers equipped with a food trough flanked on each side by retractable levers. Rats first learned to perform basic aspects of the decision-making task and to discriminate between rewards of different magnitudes. Upon completing this training, rats began training in the RDT. In this task (Fig. 1B), rats are presented with one (forced choice) or two levers (free choice) in each trial. A press on one lever resulted in the delivery of 1 food pellet, whereas a press on the other lever resulted in the delivery of 2 food pellets. Delivery of the larger reward was accompanied by possible footshock punishment (1 s), the probability of which increased across the 5 trial blocks of the session (0, 25, 50, 75, 100%). Shock intensities were initially set at 150 μA for all rats but were subsequently adjusted for each individual rat over the course of training to ensure that there was sufficient parametric space to observe changes in risk taking. Rats were trained in the RDT until behavioral stability emerged. Rats then underwent OVX followed by hormone/ER agonist treatments. Shock intensities used in the RDT post-OVX and during hormone/ER agonist treatments were the same as those used when rats achieved behavioral stability prior to surgery, and remained the same for the remainder of the experiment.

#### Progressive ratio (PR) schedule of reinforcement

Rats were tested on a PR assay to assess motivation to work for food. In this task, the number of lever presses required for the delivery of 1 food pellet increased across a session. The ratio at which the rat ceased lever pressing was identified as the rat’s breakpoint and was used as the primary outcome of this task.

#### Shock reactivity (SR) assay

To assess shock sensitivity, rats were tested in SR assay in which footshock intensities were systematically increased or decreased in response to the presence or absence of a motor response. The primary outcome was the average shock intensity at which a rat elicited a paw flinch response to footshock delivery.

### Hormone and ER agonist administration

Estradiol benzoate (EB; β-estradiol 3-benzoate, Sigma-Aldrich; 0.05 mg/mL), PPT (ERα agonist; 4,4’,4”-(4-Propyl-[1H]-pyrazole-1,3,5-triyl)trisphenol, HelloBio; 1 mg/mL), and DPN (ERβ agonist; diarylpropionitrile; HelloBio; 1 mg/mL) were dissolved in sesame oil in a warm water bath and administered subcutaneously (1 mL/kg) after daily testing. Vehicle consisted of sesame oil administered in an identical manner. This dose of EB has been shown to increase E2 levels to those seen in late-stage proestrus [40, 41]. Doses of PPT and DPN were selected due to their ability to influence effort-based decision making and anxiety-related behavior in OVX females [28, 42]. Progesterone (P4; Sigma-Aldrich; 0.6 mg/0.1 mL) was first suspended in ethanol and then combined with sesame oil to reach its final concentration in 5% v/v ethanol. Vehicle consisted of 0.1 mL of sesame oil with 5% ethanol. The dose of P4 was chosen based on prior work showing it was sufficient to induce a surge in luteinizing hormone [43, 44].

Except for treatment during the PR and SR tasks in which a between-subjects design was used, hormones and ER agonists were administered using a randomized within-subjects Latin-square design such that each rat received each treatment and vehicle. Each treatment lasted 7 days, with a minimum of 8 days between successive treatments. Injections were administered daily for 7 days and occurred approximately 1–2 h following behavioral testing. When two injections were required (e.g., EB + P4), each occurred at the same time on separate sides of the rat’s back.

### Estrous phase

Detailed descriptions of procedures related to estrous phase sampling and peripheral hormone assessments are provided in the Supplementary Material. Estrous phases were monitored via vaginal lavage to confirm that the rat’s hormonal or vaginal cytological state was consistent with their treatment group. Vaginal lavages were performed immediately after behavioral testing.

### Data analyses

A full account of statistical analyses used to analyze each dependent variable is provided in the Supplementary Material. For the RDT, the primary dependent variable was choice of the large, risky reward (i.e., risk taking). To confirm that OVX increased risk taking [21], a repeated-measures analysis of variance (RMANOVA) compared risk taking before and after OVX across all three experiments, with timepoint (pre- vs. post-OVX) and trial block (hereafter referred to as block) as within-subjects factors. For analyses of effects of hormone or ER agonist(s) on risk taking, choice performance was averaged across 7 days, beginning with day 3 of injections and ending 2 days after the last injection. This time window is consistent with prior work [21] and aligns with physiological indices of the presence of exogenous hormones in OVX rats (i.e., changes in estrous phase). These data were analyzed with a two-factor RMANOVA with treatment and block as within-subjects factors. Significant treatment effects or interactions were followed by trial-by-trial analyses to determine how treatments influenced the ability to use feedback about previous trials to guide future choices (win-stay or lose-shift behavior). These variables were analyzed using paired samples *t*-tests or RMANOVAs, with treatment as the within-subjects factor. If treatments affected choice of the large, risky reward, choice of the small, safe reward was also analyzed to determine whether choice preferences shifted together. This variable was analyzed in a manner identical to that used for choice of the large, risky reward. Effects of ER agonists on the PR and SR tasks were analyzed with a one-way ANOVA, with treatment as the between-subjects factor. Open field behavior was analyzed using an independent-samples *t*-test, with dose as the between-subjects factor. For all analyses, *p* ≤ 0.05 was considered statistically significant. If parent ANOVAs yielded main effects or significant interactions, additional ANOVAs or *t*-tests were conducted (using Bonferroni-adjusted *p*-values to correct for multiple comparisons) to identify sources of significance. Effect sizes are indicated by ${{{\rm{\eta }}}}_{p}^{2}$ for parent ANOVAs and the absolute value of Cohen’s d for *t*-tests. When mixed-effects models were used for data analysis (due to missing values), effect sizes are not reported due to software analysis limitations.

## Results

This section highlights the main results for brevity; a full exposition of the statistical results is presented in the Supplementary Material. After accounting for attrition (see Supplementary Material), the final sample sizes were n = 18 in Experiment 1, n = 37 for Experiment 2 and n = 17 for Experiment 3.

### Effects of OVX on risk taking

**Fig. 2:**
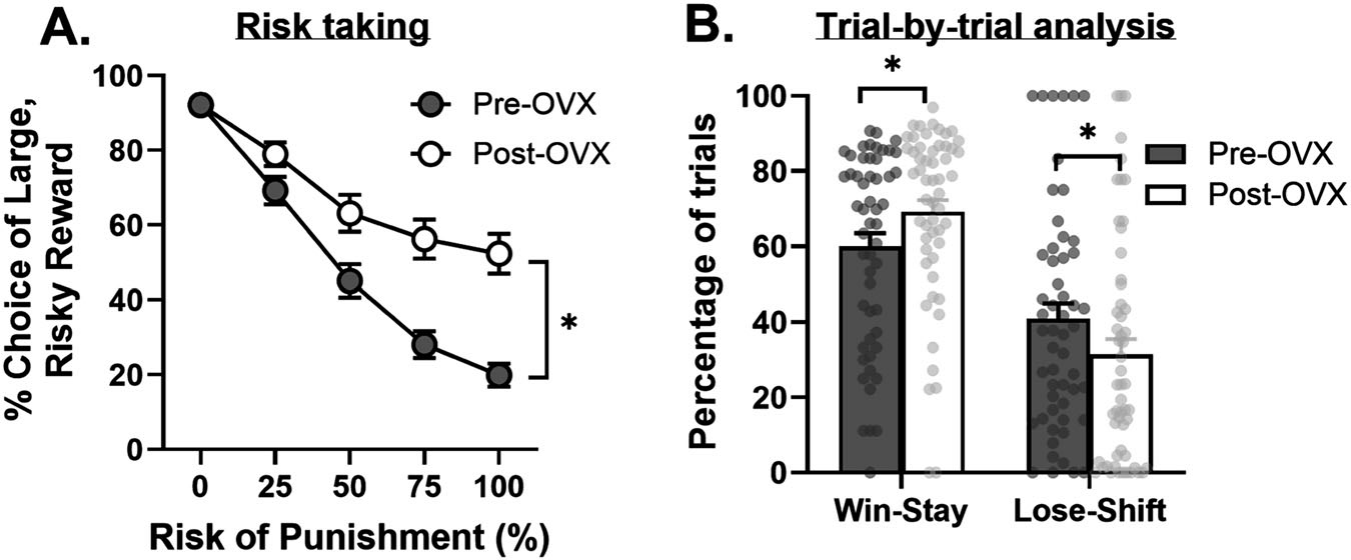
Effects of ovariectomies (OVX) on risk taking. **A** Across all experimental cohorts, there was an increase in choice of the large, risky reward (i.e., risk taking) after OVX. **B** There was a significant increase in the percentage of win-stay trials and a significant decrease in the percentage of lose-shift trials following OVX. Data are represented as mean ± standard error of the mean (SEM). Individual data points for each rat are displayed on bar graphs. Asterisks indicate *p* < 0.05. Error bars are not displayed (e.g., 0% block) when the SEM is smaller than the data point symbol.

There was no difference in the effects of OVX on risk taking between experimental cohorts [*F*(2,52) = 1.00, *p* = 0.38, ${{{\rm{\eta }}}}_{p}^{2}$ = 0.04]; thus, data were pooled for analysis of the impact of OVX on RDT measures. Consistent with prior work [21], OVX increased risk taking [Fig. 2A; time, *F*(1,54) = 46.5034, *p* < 0.01, ${{{\rm{\eta }}}}_{p}^{2}$ = 0.46; time X block, *F*(4,216) = 21.89, *p* < 0.01, ${{{\rm{\eta }}}}_{p}^{2}$ = 0.29]. This increase in choice of the large, risky reward was accompanied by a concomitant decrease in choice of the small, safe reward [*F*(1,54) = 8.26, *p* < 0.01, ${{{\rm{\eta }}}}_{p}^{2}$ = 0.13; time X block, *F*(4,216) = 8.01, *p* < 0.01, ${{{\rm{\eta }}}}_{p}^{2}$ = 0.13]. Using a mixed-effects model, analysis of win-stay and lose-shift behavior revealed a main effect of trial-type [Fig. 2B; *F*(1,50) = 31.04, *p* < 0.01] and a significant time X trial-type interaction [*F*(1,50) = 9.627, *p* < 0.01]. Post-hoc analyses showed OVX significantly increased win-stay [*t*(53) = –2.57, *p* = 0.02] and decreased lose-shift [*t*(54) = 2.63, *p* = 0.02] behavior.

### Experiment 1: effects of EB on risk taking

**Fig. 3:**
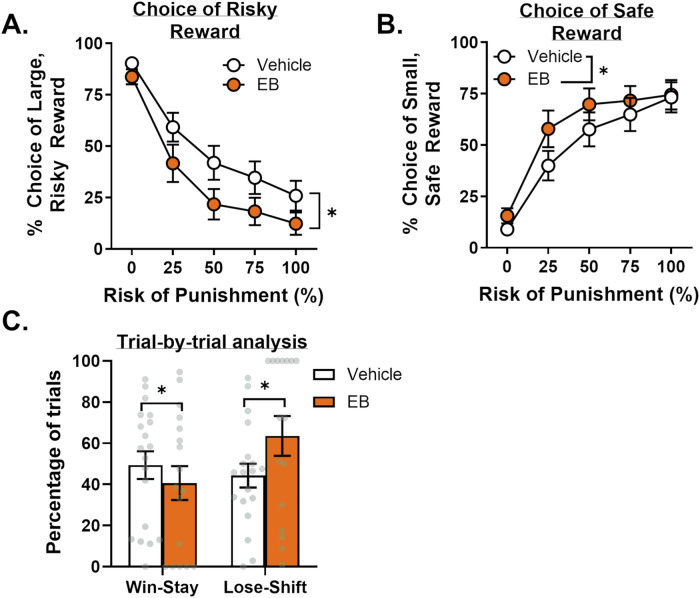
Effects of estradiol benzoate (EB) administration on risk taking in OVX females. **A** Treatment with EB decreased choice of the large, risky reward (i.e., risk taking). **B** Treatment with EB increased choice of the small, safe reward. **C** Treatment with EB decreased the percentage of win-stay trials and increased the percentage of lose-shift trials. Data are represented as mean ± standard error of the mean (SEM). Individual data points for each rat are displayed on bar graphs. Asterisks indicate *p* < 0.05. Error bars are not displayed (e.g., 0% block) when the SEM is smaller than the data point symbol.

Consistent with prior work [21], EB decreased risk taking relative to vehicle [Fig. 3A; treatment, *F*(1,18) = 34.88, *p* < 0.01, ${{{\rm{\eta }}}}_{p}^{2}$ = 0.66; treatment X block, *F*(4,72) = 2.05, *p* = 0.10, ${{{\rm{\eta }}}}_{p}^{2}$ = 0.10]. In conjunction with the decrease in choice of the large, risky reward, EB increased choice of the small, safe reward [Fig. 3B; treatment, *F*(1,18) = 11.68, *p* < 0.01, ${{{\rm{\eta }}}}_{p}^{2}$ = 0.39; treatment X block, *F*(4,72) = 3.46, *p* = 0.01, ${{{\rm{\eta }}}}_{p}^{2}$ = 0.16]. A mixed effects model yielded no main effect of treatment on win-stay and lose-shift behavior [*F*(1,18) = 1.19, *p* = 0.29]; there was, however, a significant treatment X trial-type interaction [*F*(1,13) = 17.37, *p* < 0.01]. Post-hoc analyses revealed EB increased lose-shift [Fig. 3C; *t*(19) = 3.56, *p* < 0.01] and decreased win-stay [*t*(19) = 2.32, *p* = 0.05] behavior.

### Experiment 2: effects of ER agonists on behavior

#### RDT

**Fig. 4:**
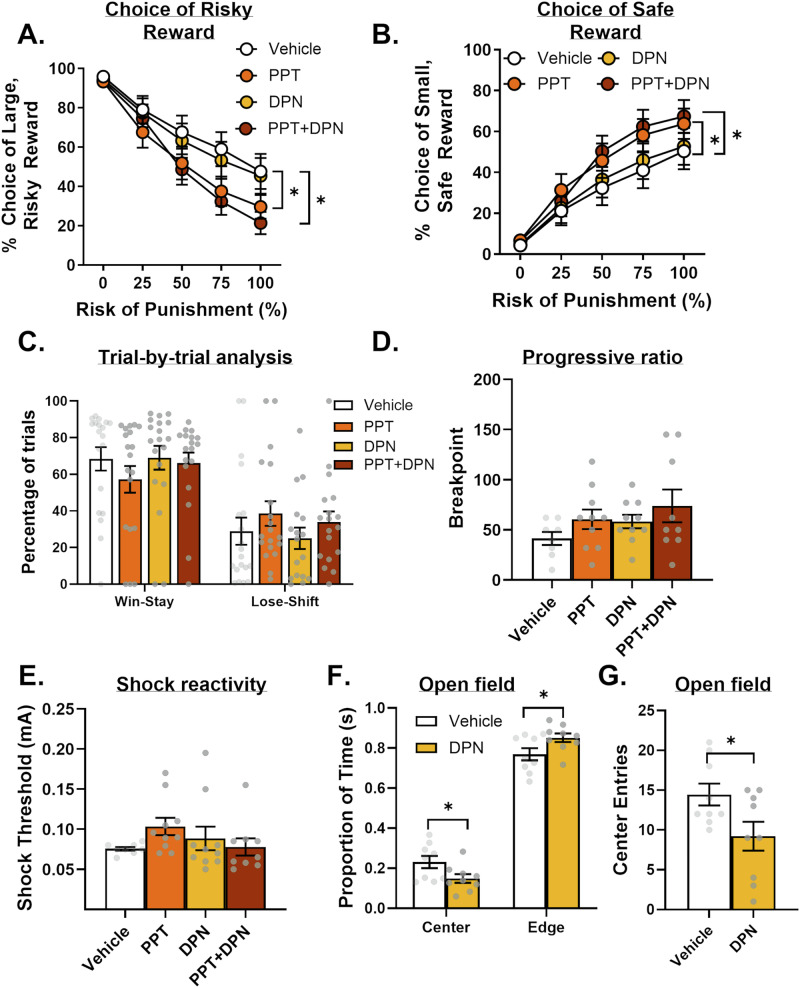
Effects of ER agonist administration on risk taking in OVX females. **A** Treatment with ERα agonist (PPT), either alone or with the ERβ agonist (DPN), decreased choice of the large, risky reward (i.e., risk taking). **B** PPT, either alone or with DPN, increased choice of the small, safe reward. **C** Treatment with ER agonists did not affect the percentage of win-stay or lose-shift trials. **D** ER agonist administration did not affect the breakpoint in the Progressive Ratio Schedule of Reinforcement assay. **E** ER agonist treatment did not affect shock reactivity thresholds. **F** DPN administration significantly decreased the time spent in the center of the open field and increased the time spent at the edges of the open field. **G** DPN administration decreased the number of entries into the center of the open field. Data are represented as mean ± standard error of the mean (SEM). Individual data points for each rat are displayed on bar graphs. Asterisks indicate *p* < 0.05. Error bars are not displayed (e.g., 0% block) when the SEM is smaller than the data point symbol.

There was a main effect of treatment [Fig. 4A, *F*(3,54) = 13.56, *p* < 0.01, ${{{\rm{\eta }}}}_{p}^{2}$ = 0.43] and a significant treatment X block interaction [*F*(12,216) = 5.82, *p* < 0.01, ${{{\rm{\eta }}}}_{p}^{2}$ = 0.24]. Post-hoc ANOVAs subsequently compared each condition (i.e., PPT, DPN, PPT + DPN) with vehicle. PPT alone or with DPN significantly decreased risk taking relative to vehicle [PPT: treatment, *F*(1,18) = 22.30, *p* < 0.01; treatment X block, *F*(4,72) = 5.43, *p* < 0.01; PPT + DPN: treatment, *F*(1,18) = 23.74, *p* < 0.01; treatment X block, *F*(4,72) = 11.33, *p* < 0.01]. Risk taking, however, did not differ between DPN and vehicle [treatment, *F*(1,18) = 1.08, *p* = 0.31; treatment X block, *F*(4,72) = 0.35, *p* = 0.84]. Analysis of choice of the small, safe reward yielded a main effect of treatment [Fig. 4B; *F*(3,54) = 8.62, *p* < 0.01, ${{{\rm{\eta }}}}_{p}^{2}$ = 0.32] and a significant treatment X block interaction [*F*(12,216) = 3.18, *p* < 0.01, ${{{\rm{\eta }}}}_{p}^{2}$ = 0.15]. Consistent with effects on choice of the large, risky reward, post-hoc analyses showed that only PPT, either alone or with DPN, increased choice of the small, safe reward [PPT: treatment, *F*(1,18) = 15.55, *p* < 0.01, ${{{\rm{\eta }}}}_{p}^{2}$ = 0.46; treatment X block, *F*(4,72) = 3.95, *p* < 0.01, ${{{\rm{\eta }}}}_{p}^{2}$ = 0.18; DPN: treatment, *F*(1,18) = 0.85, *p* = 0.37, ${{{\rm{\eta }}}}_{p}^{2}$ = 0.05; treatment X block, *F*(4,72) = 0.36, *p* = 0.84, ${{{\rm{\eta }}}}_{p}^{2}$ = 0.02; PPT + DPN: treatment, *F*(1,18) = 11.99, *p* < 0.01, ${{{\rm{\eta }}}}_{p}^{2}$ = 0.40; treatment X block, *F*(4,72) = 5.87, *p* < 0.01, ${{{\rm{\eta }}}}_{p}^{2}$ = 0.41]. A mixed-effects model found no main effect of treatment on win-stay and lose-shift behavior [*F*(3,54) = 0.07, *p* = 0.97] but did reveal a significant treatment X trial-type interaction [Fig. 4C; *F*(3,50) = 4.63, *p* < 0.01]. Subsequent post-hoc analyses, however, did not survive corrections for multiple comparisons.

#### PR task

ER agonists had no effect on breakpoint [Fig. 4D; *F*(3,33) = 1.45, *p* = 0.25, ${{{\rm{\eta }}}}_{p}^{2}$ = 0.12], number of lever presses [*F*(3,33) = 1.54, *p* = 0.22, ${{{\rm{\eta }}}}_{p}^{2}$ = 0.12] or amount of food earned [*F*(3,33) = 1.05, *p* = 0.38, ${{{\rm{\eta }}}}_{p}^{2}$ = 0.09].

#### SR assay

ER agonists did not affect rats’ shock intensity threshold [Fig. 4E; *F*(3,33) = 1.29, *p* = 0.29, ${{{\rm{\eta }}}}_{p}^{2}$ = 0.11].

#### Open field test

The absence of an effect of DPN on risk taking may be because the dose was not physiologically sufficient to have a behavioral effect. Consistent with previous work [41, 45, 46], DPN did not impact endocrine measures (Fig. S2F–H), precluding the use of these measures as positive controls for the DPN dose. The same dose of DPN, however, alters anxiety-like behavior in an open field test [47]. Consequently, a subset of females previously tested in the PR and SR assays was tested in an open field apparatus under vehicle or DPN conditions. Relative to vehicle, DPN increased time spent near the edges [Fig. 4F; *t*(16) = –2.21, *p* = 0.04, d = 0.18] and decreased time spent in the center [*t*(16) = 2.21, *p* = 0.04, d = 1.13]. DPN also decreased the number of center entries [Fig. 4G; *t*(16) = 2.29, *p* = 0.04, d = 1.08]. Collectively, these results provide a positive control for the dose of DPN.

### Experiment 3: effects of P4 on risk taking

#### RDT

**Fig. 5:**
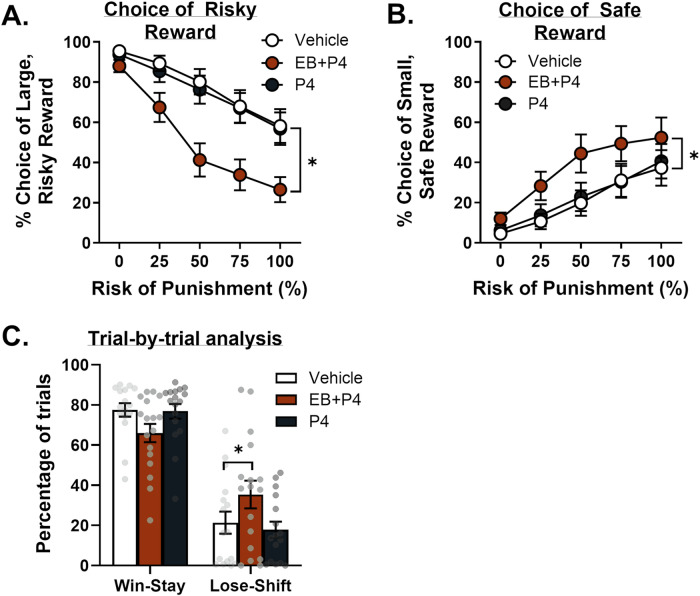
Effects of progesterone (P4) administration on risk taking in OVX females. **A** Administration of P4 alone did not affect choice of the large, risky reward (i.e., risk taking) in OVX females. In contrast, concurrent EB and P4 administration decreased risk taking in OVX females. **B** Administration of P4 alone did not alter choice of the small, safe reward in OVX females. Concurrent EB and P4 administration, however, increased choice of the small, safe reward in OVX females. **C** Administration of P4 alone did not affect the percentage of win-stay or lose-shift trials. In contrast, concurrent EB and P4 administration selectively increased the percentage of lose-shift trials in OVX females. Data are represented as mean ± standard error of the mean (SEM). Individual data points for each rat are displayed on bar graphs. Asterisks indicate *p* < 0.05. Error bars are not displayed (e.g., 0% block) when the SEM is smaller than the data point symbol.

Due to an operant box malfunction, data for the vehicle condition for one rat are missing. Consequently, behavioral data were analyzed using a mixed-effects model. There was a main effect of treatment [*F*(2,32) = 28.11, *p* < 0.01] and a treatment X block interaction [*F*(8,123) = 5.06, *p* < 0.01], with EB + P4 decreasing risk taking relative to vehicle [Fig. 5A; treatment, *F*(1,16) = 41.46, *p* < 0.001; treatment X block, *F*(4,59) = 6.70, *p* < 0.001]. In contrast, risk taking did not differ between vehicle and P4 alone [treatment, *F*(1,16) = 0.74, *p* = 0.40; treatment X block, *F*(4,59) = 0.25, *p* = 0.91]. When choice of the small, safe reward was analyzed, there was a main effect of treatment [Fig. 5B; *F*(2,32) = 11.49, *p* < 0.01] and a non-significant trend toward a treatment X block interaction [*F*(8,123) = 1.93, *p* = 0.06]. Post-hoc comparisons showed that, relative to vehicle, only EB + P4 increased choice of the small, safe reward [EB + P4: *t*(32) = 4.35, *p* < 0.01; P4: *t*(32) = 0.56, *p* > 0.10]. Analysis of win-stay and lose-shift behavior revealed no main effect of treatment [*F*(2,30) = 0.02; *p* = 0.99] but did reveal a significant treatment X trial-type interaction [*F*(2,30) = 12.49, *p* < 0.01]. Subsequent post-hoc analyses yielded a significant effect of treatment on win-stay [Fig. 5C; *F*(2,32) = 5.94, *p* < 0.01] and lose-shift [*F*(2,30) = 9.23, *p* < 0.01] behavior. Additional post-hoc comparisons revealed that EB + P4 selectively increased lose-shift behavior [*t*(16) = –3.42, *p* < 0.01]. No other post-hoc analyses survived corrections for multiple comparisons.

## Discussion

Expanding upon our previous work [22], the current study reveals the ER mechanisms by which E2 likely mediates female risk aversion. Administration of the ERα agonist PPT, either alone or with the ERβ agonist DPN, mimicked effects of EB on risk taking in OVX females. In contrast, DPN alone was ineffective in rescuing effects of OVX on risk taking. The similarity of the effects of EB and PPT on risk taking suggests that E2 may promote risk aversion in females by acting on ERα, but not ERβ. We also showed that, unlike E2, P4 does not alter risk taking in OVX females nor does it inhibit E2’s ability to attenuate the effects of OVX on risk taking. These data build upon our understanding of the role of ovarian hormones in regulating decision making involving risk of punishment by identifying the potential mechanisms by which E2 promotes risk aversion in females.

### Estradiol’s role in female risk aversion

Although hormonal fluctuations during the estrous cycle do not influence risk taking [1], the absence of ovarian hormones impacts choice behavior, causing an increase in choice of large rewards associated with risk of punishment [22]. The current study replicated effects of OVX on risk taking and did so across three separate experiments, demonstrating the reproducibility of this effect (Figs. S1A, S2A, and S3A). It further showed OVX-induced increases in risk taking were driven by augmented sensitivity to rewards and diminished sensitivity to punishment. Moreover, we examined the behavioral mechanisms driving EB’s ability to restore risk aversion in OVX females. Not only did EB decrease choice of the large, risky reward, but it also concomitantly increased choice of the small, safe reward. These data indicate that rather than causing females to entirely withhold responses, as increased omissions might suggest, EB shifted preference from risky options to safer options. Changes in choice behavior were accompanied by alterations in win-stay and lose-shift behavior. Specifically, EB decreased win-stay behavior and increased lose-shift behavior, thus restoring females’ sensitivity to rewards and punishment, respectively. Interestingly, when EB + P4 was administered, only lose-shift behavior increased. The difference in effects of EB on win-stay behavior across experiments (Experiment 1 vs. Experiment 3) could be due to inter-experimental variability. Alternatively, because EB and P4 were administered concurrently, P4 may have counteracted EB’s influence on win-stay behavior, consistent with studies showing P4 inhibits EB-induced behavioral changes in OVX females treated with EB + P4 [37, 38, 48]. Nevertheless, these findings show EB promotes risk aversion by enhancing sensitivity to punishment and, at least in the absence of P4, by reducing sensitivity to rewards.

In addition to affecting risk taking, EB increased omissions and latencies to press the large, risky lever (Fig. S1D, E). Although increased omissions could be construed as reduced motivation, EB does not affect motivation to work for food in OVX females using similar experimental parameters [21]. Rather, this effect may be another behavioral manifestation of EB’s ability to restore risk aversion in OVX females. Indeed, in addition to choice between the two levers, the option to not choose either lever (i.e., to omit) may represent a third choice available to females that could arguably be considered the safest of all options [1, 5]. Increased latency to press the large, risky lever under EB also supports a role for E2 in promoting risk-averse behavior. Alterations in latencies to press levers have been suggested to reflect changes in the incentive properties associated with available options [49–51]. Coupled with the shift in choice preference, longer latencies to press the large, risky lever may therefore signal greater apprehension associated with this lever. Consistent with this interpretation, EB increased the number of trials on which a rat initiated a trial but failed to lever press (i.e., incomplete trials). Collectively, this constellation of behavioral changes during EB treatment in OVX females provides strong support for a role of E2 in driving risk aversion in females.

In contrast to subchronic EB administration, acute EB increased risk taking in OVX females (Fig. S5). Acute effects of EB are mediated by non-genomic mechanisms, such as activation of the G-protein coupled estrogen receptor (GPER1), that support learning and memory processes [24, 52, 53]. For example, acute E2 administration prior to learning ameliorates memory deficits in OVX females [54]. Hence, acute EB may have influenced neural mechanisms involved in learning about risk and reward contingencies in the RDT that led to a transient within-session shift to the riskier reward. Future studies are needed to test this hypothesis and determine the non-genomic receptor mechanisms underlying such rapid effects of EB on risk taking.

Interestingly, EB can also enhance memory consolidation when administered after training [54–56]. Because EB administration in Experiment 1 occurred after testing, it is therefore possible that decreased risk taking observed during subchronic EB treatment was due to its effects on memory consolidation rather than on decision-making processes. This interpretation is unlikely, however, given that E2 enhances memory only when administered immediately after training; in the current study, EB was administered at least 1 h after testing, a time point beyond the window during which E2 can influence memory consolidation. Further, consolidation of long-term memories associated with RDT performance likely already occurred well before EB administration given the extensive training in the task, both before and after OVX. It is therefore more likely that subchronic EB increases risk aversion by directly affecting processes necessary for decision making involving risk of punishment (e.g., sensitivity to punishment vs. reward).

### ER mechanisms underlying E2-mediated risk aversion

Experiment 2 revealed that ERα is involved in promoting risk aversion in females. PPT administration, either alone or with DPN, mimicked effects of EB on risk taking. In contrast, DPN alone had no effect. These findings were surprising given that ERβ is involved in other aversion-based and reward-related behavior [57–59] and that an ERβ antagonist increases risk taking in intact females [22]. Several differences between studies may explain these discrepancies. For example, in contrast to the current study, Zeidan et al. administered DPN to gonadally intact females. Gonadal state alone, however, cannot fully account for discrepancies across studies, as ERβ activation can have behavioral effects in OVX females [58, 59]. A more likely explanation is that different ERs contribute to E2-mediated aversive behavior depending on whether the aversive outcome is avoidable. Indeed, ERα, but not ERβ, is necessary for learning to inhibit behavioral action to avoid an aversive outcome [60]. Similarly, risk aversion in the RDT also requires the ability to inhibit ongoing behavior to avoid possible aversive consequences. Ovariectomy disrupts this process, resulting in increased choice of punished options, and PPT ameliorates this effect. In contrast, if punishment is unavoidable, as in early trials of fear extinction [58], ERβ activation may have a more prominent role. Outside of aversively motivated behavior, ERα, albeit in conjunction with ERβ, is needed for decision making involving other costs, such as physical effort to obtain a reward [28]. When considered in this broader scope of the literature, our findings suggest a very specific role for ERα in decision making involving punishment as the cost. Finally, differences in administration parameters (e.g., treatment duration) may also explain discrepancies in results across studies.

Alternatively, the null effect of DPN may reflect inadequate dosing. Unlike EB and PPT, DPN does not affect endocrine outcomes like estrous cyclicity and uterine horn width [45, 61, 62]. Because recent work showed this dose alters anxiety-like behavior [47], rats were tested in an open field assay under DPN or vehicle to obtain a positive behavioral control for our DPN dose. Behavior in the open field was sensitive to DPN, with DPN decreasing time spent in the center. These findings confirm the behavioral efficacy of this dose of DPN and suggest the lack of an effect of DPN on risk taking likely reflects the selective activation of ERα in reducing risk taking in OVX females.

Although less established for their role in decision making, there are mechanisms by which E2 can influence cognitive function that are independent of ERα and ERβ. For example, E2 can bind to membrane-associated estrogen receptors, such as GPER1 [25], resulting in the activation of intracellular signaling cascades that support learning and memory processing [25, 52]. Importantly, however, the behavioral consequences of membrane-initiated (e.g., GPER1) cellular signaling occur within an hour of their activation. Because the change in risk taking does not emerge until day 3 of EB treatment (Fig. S1B), it is unlikely that membrane-associated estrogen receptors are activated by E2 to drive risk aversion. Given their role in rapid E2 signaling, it is more likely that membrane-associated estrogen receptors are recruited to promote *greater* risk taking as acute EB administration prior to RDT testing increased risk taking in OVX females (Fig. S5). Future studies are therefore necessary to determine whether EB’s ability to exert bidirectional control of risk taking is due to dissociable receptor and signaling mechanisms that operate on different timescales.

Interestingly, a time-course analysis of ER agonist effects on risk taking (Fig. S2B) showed that risk taking decreased on day 2 of treatment, earlier than when effects of EB appeared. Hence, PPT may have influenced behavior through ERα associated with the membrane (as opposed to ERα located in the cytoplasm or nucleus). Nevertheless, the fact that behavioral changes on day 2 resulted from injections that occurred ~21 h prior to testing suggest that recruitment of nuclear ERα is the more likely mechanism. Indeed, effects of membrane-associated ER activation dissipate within an hour of their onset [63]. This hypothesis, however, needs to be formally tested with additional studies in which ER agonists are administered prior to testing to determine whether E2 drives risk aversion in females via nuclear ERα (i.e., genomic mechanisms) or through membrane-associated ERs (e.g., non-genomic mechanisms).

### Progesterone’s role in female risk aversion

Previous work has shown that when administered alone, P4 either has no behavioral effect or leads to changes in drug-related behavior opposite to those induced by E2; when E2 and P4 are administered concurrently, P4 antagonizes the effects of E2 [32, 34, 35, 38, 48, 64]. In contrast, we show that P4 does not alter risk taking in OVX females nor does it inhibit EB’s ability to attenuate the effects of OVX on risk taking. Considered together, these findings suggest that P4’s ability to influence reward-related behavior may not extend to risk-based decision making. They also indicate that, unlike E2, P4 administration is likely insufficient to reverse OVX effects on risk taking. Prior work, however, has shown that E2 is necessary for the induction of progesterone receptor (PR) synthesis and expression in the brain and the ability of P4 to induce sexual behavior [65–67]. Hence, without EB “priming” that leads to PR expression, P4 may not be able to bind to its receptors and influence risk taking in OVX females. It is conceivable, however, that if EB and P4 were administered sequentially to mimic the endogenous surges of hormones during the estrous cycle, effects of P4 on risk taking may emerge.

### Potential neurobiological substrates of E2’s modulatory influence on risk taking

E2 likely influences risk taking in females by modulating activity of brain regions like the basolateral amygdala (BLA), which is involved in decision making involving risk of punishment. Lesions or optogenetic inhibition of the BLA increase risk taking in the RDT [49, 68], similar to effects of OVX. These parallel effects suggest female risk aversion may be dependent on E2-mediated BLA activation. In support of this, amygdala activity in rats and humans is greater during phases of the hormonal cycle in which E2 levels are high [57, 69, 70], and pharmacological reduction of E2 in women decreases BOLD signal in the amygdala [71]. E2 may regulate neural activity by binding to ERα and/or ERβ, both of which are expressed in the BLA [23]. Prior studies suggest ERβ is the primary ER through which E2 impacts BLA-mediated behavior [57, 72]. Our data suggest, however, that if E2 modulates risk aversion through ER activation in the BLA, it does so through activation of ERα. Because our manipulations were systemic and cannot speak to their locus of action in the brain, it is still conceivable that ERβ in the BLA may be involved in female risk aversion. The use of viral tools to selectively reduce expression of ERs [73–75] in the BLA would therefore allow us to determine whether E2 promotes risk aversion via activation of ERα or ERβ.

### Future directions and conclusions

Although the current work suggests that ERα activation is the primary mechanism by which E2 promotes risk aversion, additional studies are needed to directly test this hypothesis. For example, a comparison of effects of an ERα vs. ERβ antagonist in the presence of EB in OVX females would reveal the necessity of each receptor in mediating EB’s ability to drive risk aversion. This approach, however, is limited by the lack of selective ERα antagonists. Hence, the most common strategy, and thus the one employed in the current study, to examine the roles of ERs in behavior is to administer ER agonists to OVX females [28, 45, 76, 77]. Nevertheless, recent work has circumvented this pharmacological limitation and used viral-mediated selective knockdown of ERs in specific brain areas to answer similar questions about the necessity of ERs in E2-mediated behavior [73, 74]. This innovative approach would therefore provide an alternative means to test whether E2 promotes risk version via ERα activation while also identifying where in the brain this activation occurs.

In addition to affecting processes in the central nervous system that contribute to cognition, hormone treatments also impact peripheral processes, such as cardiovascular function [78] and metabolic processes [79, 80], which may influence cognitive function. Indirect support for this assertion comes from the observation that the reduction in ovarian hormones during menopause is accompanied by both cognitive decline and increased rates of cardiovascular disease [81]. Hormone treatment during menopause not only improves cognition but also reduces blood pressure and cardiovascular risk [82]. It remains unclear, however, whether the beneficial effects of treatment on cognitive function are a direct result of improved cardiovascular health. Nonetheless, it is possible that EB and/or PPT treatment may have affected peripheral processes that subsequently influenced central functions necessary for decision making. Future studies are warranted to explore this possibility and determine the implications for diseases associated with impaired cognition as well as cardiometabolic health.

In conclusion, these experiments demonstrate that E2 promotes risk aversion in females and suggest that the mechanism by which this occurs is through activation of ERα. Our findings expand upon previous work and have important implications for understanding psychiatric conditions characterized by altered risk-taking behavior, such as substance use disorder and eating disorders [83, 84]. Many of these diseases disproportionally affect women, and these sex differences are partially mediated by ovarian hormones [85, 86]. Identifying the biological mechanisms that underlie female decision making is therefore imperative to determine the basis of altered risk taking in disease states. The current study provides an initial foundation from which we can build a meaningful biological framework to explain decision making involving risk of punishment in females.

## Supplementary information

Supplementary Material

## Data Availability

Upon publication, all data will be available and searchable in the openICPSR repository. In addition to data, information about the experimental conditions and relevant subject information will be included with these files.
