# Supplementary material for: The role of ovarian hormones in risk aversion in female rats
Source: Neuropsychopharmacology. 2026 Jan 26;51(5):968–78. doi: 10.1038/s41386-026-02347-9 (PMC13013617; doi:10.1038/s41386-026-02347-9)

**SUPPLEMENTAL MATERIAL**

**Supplemental Methods**

*Subjects*

Female Long-Evans rats were purchased from Charles River Laboratories (Kingston, New York; total n=100; Experiment 1, n=20; Experiment 2, n=40; Experiment 3 and Supplemental Experiment 2, n=20; Supplemental Experiment 1, n=20) and were postnatal day 60-70 upon arrival. Rats were housed in ventilated cages with Sani-Chip bedding on a reverse light/dark cycle (lights off at 08:00 h). During food restriction, each rat’s target weight was adjusted upward by 5 g/week until they were considered fully grown (average group weight of 250 g or more), at which point they were fed 8 g of soy-free chow daily (Envigo Teklad Irradiated Global 19% Protein Extruded Rodent Diet, #2919). Wooden blocks and Nyla bones were provided as enrichment in each homecage.

*Apparatus*

Risky Decision-making Task

Behavioral testing occurred in ten identical standard operant chambers (Coulbourn Instruments, Whitehall, PA) housed in sound-attenuating cabinets (Coulbourn Instruments). Cabinets were outfitted with red lights and noise-insulating foam. Each chamber contained a centrally positioned food trough above the floor on the front wall. Food reward pellets (45 mg soy-based rodent tablets, Lab Supply, 5UTL, Northlake, TX) were delivered into the trough via a food hopper located on the back of the front wall. The food trough was equipped with photobeams to detect nosepoke entries and had a 1.12 W light bulb that was illuminated during specific task phases. Two retractable levers were located on either side of the food trough, and a 1.12 W light bulb on the back wall of the cabinet served as a house light. The chamber floor consisted of stainless-steel rods through which scrambled footshocks were delivered. A shock generator (Coulbourn Instruments) was connected to each of the chamber floors and was used to control the shock delivery and intensity. Locomotor activity was monitored in each operant chamber via a ceiling-mounted sensor that detected changes in body heat. Each chamber was connected to a computer running Graphic State 4.0 software (Coulbourn Instruments), which controlled behavioral task events and collected data in real time.

Open Field Apparatus

The open field apparatus consisted of circular open arena with a diameter of 100 cm. The walls of the arena were 56 cm high, and the floor consisted of plexiglass. A red-light source was positioned above the center of the field to ensure that the center was illuminated while the edges remained dim. A video camera was mounted overhead on a tripod to record the behavior of the rats within the apparatus. These recordings were saved on an SD card for subsequent analysis. To define the center vs. edge areas, an inner circle was drawn 15 cm from the wall, and this demarcation was used during video analysis to determine the location of the animal.

*Surgery*

All rats were ovariectomized (OVX; bilateral removal of ovaries) upon reaching behavioral stability on the RDT. Rats were anesthetized under isoflurane (1-5% in O_2_) and received subcutaneous injections of meloxicam (2 mg/kg at 1 mL/kg) and 0.9% saline (10 mL). The lower abdomen was shaved and disinfected with alcohol. Rats were transferred to a heating pad, and a sterile drape (Glad Press'n Seal) was placed over the surgical site. A small incision was made in the outer skin layer of the rat’s ventral side, and the muscle layer was blunt dissected with small surgical scissors to provide access to the peritoneal cavity. After locating the ovaries, hemostats were used to clamp the uterus below each ovary. The ovaries were removed by ligating the uterus above the hemostats with sutures. Once both ovaries were removed, the muscle layer and skin layer were closed with simple interrupted sutures. A subset of rats in Experiment 3 also received intradermal sutures to close the skin layer to reduce the likelihood of incisional dehiscence. Following surgery, rats were placed in a recovery cage with Alpha Dri bedding on a heating pad. The majority of the rats received subcutaneous injections of sustained-release (SR) buprenorphine (0.65 mg/kg) immediately after surgery; when SR buprenorphine was unavailable, rats received short-acting buprenorphine (0.03 mg/kg) every 6-8 hours for 48 hours. Rats also received meloxicam (2 mg/kg) every 24 hours for 48 hours following surgery. For at least five days after surgery, rats were given free access to food and were monitored closely for signs of health complications and to ensure successful wound closure.

*Behavioral procedures*

Shaping

Prior to training in the RDT, rats were initially shaped to perform individual components of the task. First, rats underwent magazine shaping, in which a single food pellet was dispensed into the food trough every 100 ± 40 s. Rats were required to nosepoke into the trough at least 100 times in a 30-min session to progress to the next phase of shaping. In these sessions, one of two retractable levers (left or right, counterbalanced across rats) was extended into the chamber for the duration of the test session, and rats learned that a press on the lever resulted in the delivery of a single food pellet. Once rats made at least 50 lever presses in a 30-min session, they underwent the same shaping procedure on the opposite lever and were required to meet the same passing criterion. In the final shaping session, rats learned to nosepoke into the illuminated food trough to trigger the extension of the left or right lever (randomly determined), a press on which would result in the retraction of the lever and the delivery of one food pellet. Rats were required to press each lever 30 times within the 60-min session to progress to training on the Reward Discrimination task.

Reward Discrimination Task

In this task, rats were trained to discriminate between a lever associated with the delivery of a small food reward (one food pellet) and a lever associated with the delivery of a large food reward (two food pellets). The identity of the small vs. large lever (i.e., right vs. left) was counterbalanced across rats but remained consistent throughout training on both the Reward Discrimination task and the RDT. Each 60-min session consisted of 5 blocks of 18 trials. Every 40-s trial began with the illumination of the food trough to indicate the start of the trial; a nosepoke into the trough triggered the extension of either one (forced choice trials) or both (free choice trials) levers into the chamber. If a rat failed to nosepoke within 10 s, the trough light was extinguished, and the trial was scored as an omission. A press on one lever yielded the delivery of one food pellet while a press on the other lever yielded the delivery of two food pellets. Irrespective of the lever choice, a press on either lever resulted in the retraction of lever(s) and the illumination of the food trough. If the rat did not press a lever within 15 s of their extension, lever(s) were retracted, and the trial was scored as an omission. The light in the food trough was extinguished upon food collection or after 10 s elapsed, whichever occurred first. Each block of 18 trials began with eight forced choice trials, which were followed by 10 free choice trials. Forced choice trials consisted of the extension of one lever into the chamber (four trials per lever for eight total trials) whereas free choice trials consisted of the extension of both levers into the chamber and the rats could choose freely between the two levers. In contrast to the RDT, all blocks of trials in the Reward Discrimination task were identical in design and served to instruct the rats about the structure of the task before introducing the risk of footshock punishment. To progress to the RDT, rats were required to display a preference for the large lever (>80%) for three consecutive days.

Risky Decision-making Task

The RDT was structurally identical to Reward Discrimination except that delivery of the large reward was associated with a probability of a 1-s footshock delivery that systematically increased in 25% increments over the 5 blocks of trials (0, 25, 50, 75, 100%). The forced choice trials were used to establish the risk probability in effect for each block. In these trials, the probability of footshock delivery was dependent on the outcome of previous forced choice trials on which a rat chose the large, risky lever. For example, in the 25% risk block, one and only one of the four forced trials resulted in the delivery of a footshock. In the 75% risk block, however, three out of the four forced trials resulted in footshock delivery. In contrast, the probability of footshock delivery in the free choice trials was independent of the outcome of prior free choice trials; thus, the probability of footshock delivery was equivalent across all trials in the same block of trials.

Progressive Ratio Schedule of Reinforcement

To ensure effects of ER manipulations on risk taking were not due to altered motivation to work for food, rats were tested on a Progressive Ratio Schedule of Reinforcement (PR) task under different treatment conditions. Rats were first shaped to lever press on a fixed ratio 1 (FR1) schedule, with a lever press resulting in the illumination of the trough light and the delivery of 1 food pellet. After reaching a passing criterion of at least 100 lever presses in 30 min for 2 consecutive days, rats were tested on the PR task for 7 days. Within a single PR session, the number of lever presses required to obtain a single food pellet progressed according to an arithmetic sequence (1, 3, 6, 10, 15, 21, 28, 36, 45, 55, etc.). The session ended when 10 min had passed since the delivery of the last reward.

Shock Reactivity (SR) Assay

To confirm that the effects of ER manipulations on risk taking were not due to changes in rats' sensitivity to footshock, rats were tested on a SR assay under the different treatment conditions. Rats were tested in this task 24 h after completing PR testing and therefore remained in the same treatment conditions as those assigned for the PR task. Shock reactivity for each rat was assessed in an operant chamber distinct from the one in which the rat underwent RDT and PR testing. After a 2-min habituation period, a 0.40 mA shock was delivered to reduce spontaneous locomotor activity and enable observation of subsequent motor activity at lower shock intensities. The shock intensity was then decreased to 0.05 mA, and a series of 1-s footshocks were delivered, with the intensity increasing or decreasing in increments of 0.025 mA [1–3]. Specifically, shock intensities were increased until a “flinch” (elevation of one paw) was observed, at which point the shock intensity was decreased. Trials progressed in this way until at least three flinches were observed; these three shock intensities were averaged together to produce a mean shock reactivity threshold for each rat.

Open Field Test

As a positive control for DPN treatment, a subset of rats was tested in the open field test to assess anxiety-like behavior. Each rat was placed on the edge of the circular testing apparatus facing the center, and the entire 5-min session was video recorded for later analysis. The floor of the open field was cleaned between each rat with a 0.72% Nolvasan solution.

*Hormone and ER agonist administration*

Hormone (i.e., EB and/or P4) and ER agonists were injected subcutaneously 1-2 h after behavioral testing. For treatment conditions in which rats received two compounds (e.g., PPT+DPN; EB+P4), the different agonists or hormones were administered as separate injections on opposite sides of the rat’s back. For testing in the RDT, assignment of treatment group was randomly determined prior to the start of treatment using a within-subjects Latin-Square design. In contrast, ER agonists were administered using a between-subjects design during testing in the PR and SR assays and collection of endpoint measures. Vaginal lavages were performed daily during each treatment regimen to confirm that the physiological state of the rat was consistent with the treatment group. Between successive treatments, vaginal lavage was used to ensure that all rats returned to a vaginal cytological state associated with OVX (i.e., persistent diestrus; see *Estrous phase* in *Data analysis*). Between successive treatments, performance in the RDT was monitored and analyzed to confirm that behavior had re-stabilized and was no different from rats’ original baseline (i.e., pre-treatment).

*Estrous phase monitoring*

A small volume (~50uL) of saline was lavaged into the rat’s vagina and collected on a glass slide. Samples were read immediately with a light microscope at 10x magnification. The estrous phase was identified based on the cellular cytology present in the sample [4–6]. Briefly, proestrus was characterized by the predominance of nucleated epithelial cells, whereas estrus was characterized by the predominance of cornified epithelial cells. Metestrus was defined as a mixture of nucleated cells, cornified cells, and leukocytes or neutrophils, and diestrus consisted of mostly leukocytes. Metestrus (M) and diestrus (D) are estrous phases associated with lower E2 and P4 levels, while proestrus (P) and estrus (E) are phases associated with higher circulating levels of these hormones [6–9].

*Endpoint measurements*

Prior to sacrifice, rats were treated with EB (Experiment 1), ER agonists (Experiment 2), P4 (Experiment 3) or vehicle (all Experiments) according to a between-subjects design for at least five days. At the time of sacrifice, rats were lightly anesthetized with isoflurane and then rapidly decapitated. Trunk blood was collected for serum assays and uterine horn width was measured.

Serum collection

To confirm that hormone treatments sufficiently increased circulating E2 and P4 to levels that were physiologically relevant, serum was collected for analysis in Experiments 1 and 3. Serum was also collected from rats in Experiment 2 even though serum levels of E2 and P4 were not expected to change under ER agonist conditions. Trunk blood was collected and then stored on ice until the time of serum extraction. Samples sat for a minimum of 30 min at room temperature to allow blood to clot and were then spun in a centrifuge for at least 5 min at 1500 g at room temperature. The supernatant was removed and pipetted into a separate tube and frozen at -80°C until the samples were used for assays.

Serum analysis of E2 and P4

Serum E2 concentrations were measured in duplicate using an Ultra-Sensitive Estradiol Radioimmunoassay (RIA) kit (DSL-4800; Beckman Coulter, Brea, CA) with intraassay variabilities of 5.25% in Experiment 1, 10.21% and 2.73% in Experiment 2, and 9.04% in Supplemental Experiment 1. P4 was measured in duplicate serum samples at a 1:600 dilution using a Progesterone Enzyme-linked immunosorbent assay (ELISA) kit (582601; Cayman Chemical, Ann Arbor, MI) with intraassay variabilities of 4.96% in Experiment 1, 7.96% and 10.83% in Experiment 2, and 5.34% in Supplemental Experiment 1.

Uterine horn measurements

The width of each uterine horn was measured in millimeters. These measurements were made as close to the meeting point of the cervix as possible; the diameters of the left and right horn were averaged together to produce a mean uterine horn width measurement for each rat.

*Data Analysis*

Data were extracted and analyzed using customized Graphic State 4.0 analysis templates for each behavioral task. Statistical analyses were conducted with SPSS 29.0 and figures were created with GraphPad Prism 10.0.

Risky Decision-making Task

The primary dependent variable in the RDT was the percentage of free choice trials in each block on which a rat chose the large, risky lever. To determine behavioral stability on the RDT, a repeated-measures ANOVA (RMANOVA) was used to compare percent choice in each of the 5 blocks of trials over 3 consecutive days. Behavioral stability was achieved when there was a main effect of trial block (hereafter referred to as block), indicating that rats discounted the large reward as the risk of punishment increased, but no main effect of day and no significant interaction between day and block. To confirm that OVX increased percent choice of the large, risky reward (hereafter referred to as risk taking), stable performance in the RDT was compared before and after OVX using a RMANOVA, with timepoint (pre vs. post-OVX) and block as within-subjects factors. Consistent with our prior work [3], analysis of the effects of treatment began on the first day on which physiological measures of treatment were detected (changes in the estrous phase; day 3 of injections) and ended 2 days after injections concluded, a timepoint that coincided with when physiological measures of treatment (changes in estrous phase) were no longer observed. Percent choice of the large, risky reward in each block was averaged across this period of time. A RMANOVA was then used to compare treatment conditions with vehicle, with treatment and block as within-subjects factors. If treatments significantly impacted choice of the large, risky reward, additional analyses were conducted to determine whether choice of the small, safe reward changed in tandem with choice of the large, risky reward. These analyses were conducted in a manner identical to analyses used for choice of the large, risky reward.

If there were significant effects of treatment on choice of the large, risky reward, trial-by-trial analyses were conducted to determine how outcomes from the previous trial affected choice on the subsequent trial. Trials were categorized as either “win-stay” or “lose-shift.” A win-stay trial was defined as a trial on which a rat chose the large, risky lever after choosing this same lever on the previous trial and receiving a large reward without punishment. In contrast, a lose-shift trial was defined as a trial on which a rat chose the small, safe lever after choosing the large, risky lever on the previous trial and receiving both a large reward and footshock. While win-stay trials provided a measure of sensitivity to reward, lose-shift trials served as a proxy for sensitivity to punishment [10–12]. Win-stay behavior was calculated by dividing the number of trials on which a rat chose the large, risky lever after receipt of the large, unpunished reward in the preceding trial by the total number of free choice trials on which the rat received the large, unpunished reward. Lose-shift behavior was calculated by dividing the number of trials on which a rat shifted choice to the small, safe lever after receipt of the large, punished reward by the total number of free choice trials on which the rat received the large, punished reward. A mixed-effects model was used to compare the percentage of win-stay and lose-shift trials before and after OVX or across treatment conditions. This method of analysis was chosen over a repeated-measures ANOVA due to the fact that several rats had missing data for certain trial types (e.g., a rat never exhibited win-stay behavior under EB treatment). In these models, rats were entered as random factors whereas trial-type and timepoint or treatment condition were entered as fixed factors.

Ancillary analyses were conducted on other behavioral measures, including latencies to press levers in the forced choice trials, locomotor activity, and percentage of omissions and incomplete trials. Latency to press each lever during a trial was defined as the amount of time between the initiation of a trial (i.e., a nosepoke) and a lever press. Latencies were analyzed only for forced choice trials to ensure there was a sufficient number of trials for each lever (i.e., in certain blocks of free choice trials, rats would display 100% preference for one lever over the other, precluding the ability to assess latencies for that lever in that block of trials). Latencies to press levers were analyzed with a RMANOVA, with timepoint or treatment condition, lever identity (small/safe vs. large/risky), and block as within-subjects factors. Locomotor activity was defined as an arbitrary value representing the change in energy calculated by an infrared detector located on the ceiling of each operant chamber and positioned directly over the center of the chamber. Baseline locomotor activity was averaged across the intertrial intervals of all blocks. Locomotor activity during shock delivery was calculated by averaging locomotor activity during the delivery of the 1-s footshock across blocks 2-5 (i.e., all blocks in which there was a risk of footshock delivery). Effects of OVX on baseline locomotor activity and locomotor activity during shock delivery were assessed using a paired-samples *t*-test, with timepoint as the within-subjects factor. To determine the effects of hormone or ER agonist treatment on locomotion, locomotor activity was averaged across the 7 days of testing that corresponded with physiological indices of treatment efficacy (i.e., from day three of injections to two days after injections ceased). These data were then analyzed with a paired-samples *t*-test (Experiment 1) or RMANOVA (Experiment 2), with treatment condition as the within-subjects factor. Omitted trials (otherwise referred to as omissions) were defined as the percentage of free choice trials on which a rat failed initiate a trial. Incomplete trials were operationalized as the percentage of trials on which rats initiated a free choice trial with a nosepoke but failed to lever press. The percentage of omissions was calculated by dividing the number of omitted trials by the total number of free choice trials (and multiplied by 100). The percentage of incomplete trials was calculated by dividing the number of incomplete trials by the number of completed free choice trials (and multiplied by 100). These dependent variables were analyzed with a paired-samples t-test in Experiment 1 and a RMANOVA in Experiments 2 and 3, with treatment condition as the within-subjects factor in each analysis.

Estrous phase

Estrous phases were monitored prior to and during hormone or ER agonist treatment. Prior to treatments, the efficacy of OVX was confirmed when rats were no longer cycling through different phases and remained in permanent metestrus/diestrus. In Experiments 1 and 3, the number of days spent in proestrus/estrus (hormonal P/E; hereafter referred to as a high hormone state) or metestrus/diestrus (hormonal M/D; hereafter referred to as a low hormone state) was quantified for each treatment condition. Changes in phases were thus indicative of changes in hormone states. In Experiment 2, differences in the characteristics of vaginal cell cytology were used to confirm physiological efficacy of ER agonist treatment (versus vehicle treatment). Changes in the distribution of cell types reflected the effects of ER activation in reproductive tissue (as opposed to reflecting circulating hormone levels) [13]. The presence of nucleated and anucleated cornified cells was classified as a cytological P/E state whereas the presence of a mix of cornified cells and leukocytes or predominantly leukocytes was classified as a cytological M/D state. In all experiments, a RMANOVA was used to compare the number of days in each state (hormonal or cytological) between the different treatment conditions, with state (P/E vs. M/D) and treatment condition as within-subjects factors. It is important to note that the statistics analyzing time spent in high vs. low hormone states have a proportional relationship.

Uterine horn measurements

Treatment condition was included as a between-subjects factor in all analyses of endpoint measures because this round of treatment ended in sacrifice for blood collection. In Experiment 1, uterine horn widths were analyzed with an independent samples *t*-test. In Experiments 2 and 3, uterine horn widths were analyzed with a one-way ANOVA.

Serum Assays

Similar to analysis of uterine horn widths, serum levels of E2 and P4 were analyzed with an independent samples *t*-test in Experiment 1. In Experiments 2 and 3, serum levels were analyzed with a one-way ANOVA. In all analyses, treatment condition was included as the between-subjects factor.

Progressive Ratio Schedule of Reinforcement

The main dependent variables for the PR task were 1) the number of lever presses, 2) the breakpoint ratio (or the ratio at which the rat ceased lever pressing), and 3) the number of rewards earned. These measures were averaged across days 3 through 7 (5 days) of ER agonist treatment, and these means were compared between ER agonist treatment conditions using one-way ANOVAs with treatment as the between-subjects factor.

Shock Reactivity Assay

On the shock reactivity assay, the average shock reactivity threshold was compared between vehicle and the different ER agonist treatment conditions using a one-way ANOVA with treatment as the between-subjects factor.

Open Field Test

The main dependent variables in the open field test were 1) the percentage of time spent in the center space, 2) the percentage of time spent in the edge space, and 3) the number of entries into the center space. The effect of the ERβ agonist DPN on these variables was analyzed using independent samples *t*-tests, with treatment (vehicle vs. DPN) as the between-subjects factor.

**Supplemental Results**

In Experiment 1, one rat died due to surgical complications, and one rat was excluded due to inability to acquire the task, yielding a final sample size of 18. In Experiment 2, three rats were euthanized due to surgical complications, resulting in a final sample size of 37. In Experiment 3 and Supplemental Experiment 2, one rat died overnight following surgery, and two rats were euthanized due to surgical complications, resulting in a final sample size of 17. In Supplemental Experiment 1, one rat was euthanized because of surgical complications, yielding a final sample size of 19.

*Experiment 1: Effects of EB on risk taking*

Risky Decision-making Task

Rats were trained in the RDT for 59 days, at which point behavioral stability was achieved. After OVX, rats required 20 days to once again exhibit stable performance in the RDT. Relative to performance prior to OVX, there was a significant increase in choice of the large, risky reward [Figure S1A; time, *F*(1,18)=7.26, *p*=0.02, $ƞ_{p}^{2}$=0.20; time X block, *F*(4,72)=3.89, *p*<0.01, $ƞ_{p}^{2}$=0.18]. After OVX, there was a near-significant decrease in choice of the small, safe reward [time, *F*(1,4)=4.13, *p*=0.06, $ƞ_{p}^{2}$=0.0.19; time X block, *F*(4,72)=2.04, *p*=0.10, $ƞ_{p}^{2}$=0.0.10]

To examine whether effects of EB on risk taking could be due to the genomic effects of EB, a time-course analysis of choice behavior was conducted. Choice of the large, risky reward was averaged across blocks 2 through 5 (i.e., trial blocks in which there was risk of punishment) and compared between treatment conditions across the treatment period. There was a main effect of treatment [*F*(1,18)=15.15, *p*<0.01, $ƞ_{p}^{2}$=0.46] and a significant treatment X day interaction [*F*(7,126)=4.02, *p*<0.01, $ƞ_{p}^{2}$=0.18]. Inspection of Figure S1B suggests that this interaction was driven by a decrease in choice of the large, risky reward on day 3 that persisted through the end of the treatment period. This effect is consistent with the time course of changes in the hormonal states under these treatment conditions [3].

Consistent with previous work [14], latencies to press the large, risky lever were significantly longer than those to press the small, safe lever [lever identity, *F*(1,17)=45.37, *p*<0.01, $ƞ_{p}^{2}$=0.73; lever identity X block, *F*(4,68)=46.51, *p*<0.01, $ƞ_{p}^{2}$=0.73]. A three-factor RMANOVA revealed that EB significantly affected this behavioral measure, with main effect of treatment [Figure S1C and S1D; *F*(1,17)=24.23, *p*<0.01, $ƞ_{p}^{2}$=0.59] as well as treatment X lever identity [*F*(1,17)=5.71, *p*=0.03, $ƞ_{p}^{2}$=0.25], treatment X block [*F*(4,68)=6.41, *p*<0.01, $ƞ_{p}^{2}$=0.27] and treatment X lever identity X block [*F*(4,68)=3.77, *p*<0.01, $ƞ_{p}^{2}$=0.18] interactions. These interactions suggested that EB selectively affected latencies to press one lever over the other. Post-hoc analyses were therefore conducted to compare latencies between treatment conditions for each lever separately. These analyses revealed that, relative to vehicle, EB increased latencies to press the small, safe lever [treatment, *F*(1,18)=9.90, *p*<0.01, $ƞ_{p}^{2}$=0.36, treatment X block, *F*(4,72) = 2.65, *p*=0.04, $ƞ_{p}^{2}$=0.13] as well as the large, risky lever [treatment, *F*(1,17)=14.58, *p*<0.01; treatment X block, *F*(4,68)=5.18, *p*<0.01]. In addition to the effects on latencies, EB decreased baseline locomotor activity [*t*(16)=6.95, *p*<0.01, d=1.69; Supplemental Table 1]. There was, however, no effect of EB on locomotor activity during shock delivery [*t*(13)=0.52, *p*=0.62, d=0.14; Supplemental Table 1]. Not only did EB increase the percentage of omissions [Figure S1E; *t*(18)=-4.57, *p*<0.01, d=1.05], it also increased the number of incomplete trials [i.e., number of initiated but not completed trials; *t*(18)=-2.18, *p*=0.04, d=0.50; vehicle: 0.03 (±0.03); EB: 0.40 (±0.17)].

Estrous phase

Prior to EB treatment, all OVX females were in a persistent cytological M/D state, consistent with the absence of circulating ovarian hormones. Although there was no main effect of EB treatment on the number of days spent in high vs. low hormone states [*F*(1,18)=1.73, *p*=0.21, $ƞ_{p}^{2}$=0.09], there was a significant treatment X hormone state interaction [Figure S1F; *F*(1,18)=99.62, *p*<0.01, $ƞ_{p}^{2}$=0.85]. Subsequent post-hoc *t*-tests revealed that EB increased the number of days spent in the high hormone state [*t*(19)=-7.28, *p*<0.01] and decreased the number of days spent in the low hormone state [*t*(19)=6.84, *p*<0.01].

Serum Assays

EB treatment increased serum levels of E2 [Figure S1G; *t*(6)=7.75, *p*<0.01, d=5.48] but did not alter serum levels of P4 [Figure S1G; *t*(6)=1.57, *p*=0.17, d=1.11].

Uterine Horn Width

Treatment with EB increased uterine horn width [Figure S1H; *t*(6)=5.82, *p*<0.01, d=4.11], which is consistent with prior work [15–19].

*Experiment 2: Effects of ER agonists on behavior*

Risky Decision-making Task

Rats were trained in the RDT for 63 days, at which point behavioral stability was established. After OVX, rats required 14 days to re-establish behavioral stability in the RDT. There was a significant increase in choice of the large, risky reward following OVX [Figure S2A; time, *F*(1,18)=21.23, *p*<0.01, $ƞ_{p}^{2}$=0.54; time X block, *F*(4,72)=11.40, *p*<0.01, $ƞ_{p}^{2}$=0.39] and a significant decrease in choice of the small, safe reward [time, *F*(1,18)=3.34, *p*=0.08, $ƞ_{p}^{2}$=0.16; time X block, *F*(4,72)=4.07, *p*<0.01, $ƞ_{p}^{2}$=0.18].

Similar to Experiment 1, average choice of the large, reward was compared between treatment conditions across the treatment period. As shown in Figure S2B, effects of PPT administration, either alone or with DPN, emerged on day 2 and persisted through the end of treatment [treatment, F(3,54)=15.47, *p*<0.01, $ƞ_{p}^{2}$=0.46; treatment X day, *F*(21,378)=2.35, *p*<0.01, $ƞ_{p}^{2}$=0.12]. Post-hoc comparisons confirmed that this effect was specific to PPT conditions [PPT: treatment, *F*(1,18)=19.65, *p*<0.01, $ƞ_{p}^{2}$=0.52; treatment X day, F(7,126)=2.75, p=0.01, $ƞ_{p}^{2}$=0.13; DPN: treatment, *F*(1,18)=0.77, *p*=0.39, $ƞ_{p}^{2}$=0.04; treatment X day, *F*(7,126)=0.84, *p*=0.56, $ƞ_{p}^{2}$=0.05; PPT+DPN: treatment, *F*(1,18)=36.40, *p*<0.01, $ƞ_{p}^{2}$=0.67; treatment X day, *F*(7,126)=3.47, *p*<0.01, $ƞ_{p}^{2}$=0.16].

Analyses of latencies to press levers revealed that ER agonists had no effect on this behavioral measure [Figure S2C and S2D; treatment, *F*(3,54)=0.78, *p*=0.51, $ƞ_{p}^{2}$=0.04; treatment X lever identity, *F*(3,54)=0.43, *p*=0.74, $ƞ_{p}^{2}$=0.02; treatment X lever identity X block, *F*(12,216)=0.72, *p*=0.73, $ƞ_{p}^{2}$=0.04] nor did they alter locomotor activity at baseline [*F*(3,45)=0.12, *p*=0.95, $ƞ_{p}^{2}$<0.01; Supplemental Table 1] or during shock delivery [*F*(3,39)=1.95, *p*=0.14, $ƞ_{p}^{2}$=0.13; Supplemental Table 1]. There was, however, a main effect of treatment on the percentage of omissions [Figure S2E; *F*(3,54)=10.68, *p*<0.01, $ƞ_{p}^{2}$=0.37]. Post-hoc analyses revealed that, consistent with the effects on choice behavior, PPT alone [*t*(18)=-3.95, *p*<0.01, d=0.91] or with DPN [*t*(18)=-3.83, *p*<0.01, d=0.88], increased omissions. In contrast, DPN did not affect omissions relative to vehicle [*t*(18) =-0.58, *p*=0.57, d=0.13]. In contrast to the effects on percentage of omissions, there was no effect of ER agonists on the percentage of incomplete trials [*F*(3,54)=0.37, *p*=0.77, $ƞ_{p}^{2}$=0.02; vehicle: 0.06 (±0.04), PPT: 0.08 (±0.06), DPN: 0.12 (±0.09), PPT+DPN: 0.04 (±0.03)].

Estrous phase

Prior to ER agonist treatment, all OVX rats were in a constant cytological M/D state. Although there was no main effect of treatment on the number of days spent in cytological P/E vs. cytological M/D [*F*(3, 54)=1.00, *p*=0.40, $ƞ_{p}^{2}$=0.05], there was a significant interaction between treatment and state [Figure S2F; *F*(3, 54)=185.10, *p*<0.01, $ƞ_{p}^{2}$=0.91]. Post-hoc analyses were subsequently used to determine the source of this significant interaction. Administration of PPT alone or with DPN increased the number of days in the cytological P/E state [PPT: *t*(19)=12.38, *p*<0.01; PPT+DPN: *t*(19)=11.98, *p*<0.01] and decreased the number of days spent in the cytological M/D state [PPT: *t*(19)=12.78, *p*<0.01; PPT+DPN: *t*(19)=11.98, *p*<0.01]. In contrast, the number of days spent in either cytological state did not differ between vehicle and DPN [P/E: *t*(19)=1.07, *p*=0.87; M/D: *t*(19)=1.07, *p*=0.87].

Serum Assays

Administration of ER agonists did not alter serum levels of E2 [Figure S2G; *F*(3,33)=0.45, *p*=0.72, $ƞ_{p}^{2}$=0.04] or P4 [Figure S2G; *F*(3,33)=1.37, *p*=0.27, $ƞ_{p}^{2}$=0.11].

Uterine Horn Width

Consistent with the effects of ER agonist treatment on cytological states, there was a main effect of ER agonist treatment on uterine horn width [Figure S2H; *F*(3,32)=53.64, *p*<0.01, $ƞ_{p}^{2}$=0.83]. Post-hoc analyses revealed that this effect was driven by the action of the ERα agonist. Uterine horn width was increased in both PPT [*t*(8)=8.50, *p*<0.01] and PPT+DPN conditions [*t*(8)=9.72, *p*<0.01]. In contrast, after correcting for multiple comparisons, DPN did not alter uterine horn width compared to vehicle [Figure S2H; *t*(8)=0.87, *p*>0.10].

*Experiment 3: Effects of P4 on risk taking*

Risky Decision-making Task

Rats were trained in the RDT for 46 days, at which point behavioral stability was achieved. After OVX, rats required 7 days to re-establish behavioral stability in the RDT. After OVX, there was a significant increase in choice of the large, risky reward [Figure S3A; time, *F*(1,16)=22.90, *p*<0.01, $ƞ_{p}^{2}$=0.59; time X block, *F*(4,64)=12.31, *p*<0.01, $ƞ_{p}^{2}$=0.44] and a significant decrease in choice of the small, safe reward [time, *F*(1,16)=10.62, *p*<0.01, $ƞ_{p}^{2}$=0.40; time X block, *F*(4,64)=5.35, *p*<0.01, $ƞ_{p}^{2}$=0.25].

Analysis of choice of the large, risky reward was compared between treatment conditions across the treatment period with a mixed effects model. There was a main effect of treatment [*F*(2,32)=15.13, *p*<0.01] and a significant treatment X day interaction [*F*(14,216)=12.88, *p*<0.01]. As shown in Figure S3B, females decreased their choice of the large, risky reward under conditions of EB+P4, but not P4 alone [vehicle vs. EB+P4: treatment, *F*(1,16)=23.19, *p*<0.01; treatment X day, *F*(7,104)=13.27, *p*<0.01; vehicle vs. P4: treatment, *F*(1,16)=1.09, *p*=0.31; treatment X day, *F*(7,104)=1.77, *p*=0.10]. Consistent with Experiment 1, this decrease appeared on day 3 of treatment.

Latencies to press levers differed across treatment conditions [*F*(2,32)=3.21, *p*=0.05, $ƞ_{p}^{2}$=0.17], but this effect did not depend on the identity of the lever [Figure S3C and S3D; treatment X lever identity, *F*(2,32)=2.42, *p*=0.11, $ƞ_{p}^{2}$=0.13; treatment X lever identity X block, *F*(8,128)=1.01, *p*=0.43, $ƞ_{p}^{2}$=0.06]. Latencies to press levers were then compared between vehicle and each treatment. Although it appeared that the EB+P4 increased latencies, these analyses did not survive corrections for multiple comparisons. There was a main effect of treatment on the percentage of omissions [*F*(2,32)=28.01, *p*<0.01, $ƞ_{p}^{2}$=0.64]. Post-hoc analyses revealed that EB+P4 significantly increased omissions [Figure S3D; *t*(16)=-6.10, *p*<0.01, d=1.48]; in contrast, P4 had no effect on omissions [*t*(16)=-0.64, *p*=0.53, d=0.16]. Hormone treatment did not significantly affect the number of incomplete trials [*F*(2, 32)=0.34, *p*=0.72, $ƞ_{p}^{2}$=0.02; vehicle: 0.09(±0.04), EB+P4: 0.14(±0.07), P4: 0.08(±0.08)]. Malfunctions in the locomotor sensors precluded the acquisition and analysis of baseline locomotor activity and locomotor activity during the shock delivery in this Experiment.

Estrous phase

Following OVX, rats were in a persistent cytological M/D state. Analysis of the effects of treatment on the number of days spent in high vs. low hormone states revealed that there was not a main effect of treatment [*F*(2,30)=2.90, *p*=0.07, $ƞ_{p}^{2}$=0.16], but there was a significant interaction between treatment condition and hormone state [Figure S3E; *F*(2,30)=75.45, *p*<0.01]. This interaction was driven by a selective effect of EB+P4 [EB+P4: treatment, *F*(1,15)=4.29, *p*=0.05; treatment X hormone state, *F*(1,15)=70.13, *p*<0.01; P4: treatment, *F*(1,15)=3.30, *p*=0.09; treatment X hormone state, *F*(1,15)=0.35, *p*=0.57]. Specifically, EB+P4 increased the number of days spent in the high hormone state [*t*(16)=10.72, *p*<0.01] and decreased the number of days spent in the low hormone state [*t*(16)=9.22, *p*<0.01]. The absence of an effect of P4 in OVX females is consistent with previous work showing that P4 administration suppresses estrous cycling in intact females [20].

**Supplemental Experiments:**

*Supplemental Experiment 1: Validation of progesterone (P4) dose with serum hormone levels*

To determine the dose of P4 required to elevate circulating levels of this hormone, rats (n=20) were ovariectomized, received injections of two different doses of P4 (Sigma-Aldrich; 0.5 mg/kg, 0.6 mg/0.1mL) or vehicle, and then euthanized for serum collection and uterine horn measurements. For the 0.5 mg/kg dose, P4 (0.5 mg/mL) was dissolved in sesame oil in a warm water bath and administered at a volume of 1 mL/kg. This dose of P4 was selected based on previous work that showed it altered impulsivity and depressive-like behavior [21–23]. For the 0.6 mg dose, P4 was first suspended in ethanol and then combined with sesame oil for a final concentration of 0.6 mg/0.1 mL in 5% v/v ethanol. The solution was stirred and warmed to ensure P4 remained in solution. Vehicle consisted of 0.1 mL of sesame oil with 5% ethanol. This higher dose of P4 was chosen based on prior work in which it effectively induced a surge in luteinizing hormone [24,25]

A one-way ANOVA revealed a main effect of dose on P4 serum levels [Figure S4A; *F*(2,13)=29.66, *p*<0.01, $ƞ_{p}^{2}$=0.82]. Post-hoc analyses confirmed that only the 0.6 mg/0.1 mL dose effectively increased P4 serum levels (vehicle vs. 0.6 mg/mL: *t*(13)=7.12, *p*<0.01; vehicle vs. 0.5 mg/kg: *t*(13)=0.54, *p*>0.99. In contrast, neither dose of P4 affected E2 serum levels [Figure S4B; *F*(2,13)=1.35, *p*=0.29, $ƞ_{p}^{2}$=0.17]. Additionally, uterine horn width was not altered by either dose of P4 [Figure S4C; *F*(2,13)=0.63, *p*=0.55, $ƞ_{p}^{2}$=0.09]. Based on these data, the dose of 0.6 mg/0.1 mL was chosen for Experiment 3.

*Supplemental Experiment 2: Effects of acute EB administration on risk taking*

To assess the non-genomic effects of E2 on risk taking, rats (n=17) that were used in Experiment 3 were re-trained in the RDT after completing P4 administration until stability was achieved. Using a within-subjects design, rats received subcutaneous injections of vehicle (sesame oil) or EB (0.05 mg/kg administered at a volume of 1mL/kg) 30 min prior to testing in the RDT, with two washout days between successive injections. An RMANOVA revealed a main effect of EB, with acute EB increasing risk taking relative to vehicle [Figure S5A; *F*(1,16)=7.22, *p*=0.02, $ƞ_{p}^{2}$=0.31]. There was, however, no EB X block interaction [*F*(4,64)=0.62, *p*=0.65, $ƞ_{p}^{2}$=0.04]. In conjunction with the increase in choice of the large, risky reward, acute EB significantly decreased choice of the small, safe reward [Figure S5B; EB, *F*(1,16)=5.41, *p*=0.03, $ƞ_{p}^{2}$=0.26; EB X block, *F*(4,64)=1.21, *p*=0.32, $ƞ_{p}^{2}$=0.07]. Analysis of win-stay and lose-shift behavior did not yield a main effect of EB [*F*(1,16)=1.27, *p*=0.27], but there was a significant EB X trial-type interaction [Figure S5C; *F*(1,13)=5.55, *p*=0.03]. Post-hoc analyses revealed EB significantly increased the percentage of win-stay trials [*t*(17)=2.14, *p*=0.04] without altering the percentage of lose-shift trials [*t*(16)=0.17, *p*=0.86]. Acute EB did not affect latencies to press levers [Figure S5D and S5E; EB, *F*(1,16)=3.10, *p*=0.10, $ƞ_{p}^{2}$=0.16; EB X lever identity, *F*(1,16)=0.62, *p*=0.44, $ƞ_{p}^{2}$=0.04; EB X block, *F*(4,64)=0.61, *p*=0.66, $ƞ_{p}^{2}$=0.04; dose X lever identity X block, *F*(4,64)=1.45, *p*=0.23, $ƞ_{p}^{2}$=0.08]. Finally, and consistent with an increase in risk taking, acute EB decreased the percentage of omissions [Figure S5F; *t*(16)=2.60, *p*=0.02, d=0.63]. There were no incomplete trials under acute vehicle of EB conditions. Like Experiment 3, malfunctions in the locomotor sensors prevented the ability to acquire and analyze locomotor activity in this Experiment. Collectively, these data suggest that in contrast to the long-term genomic actions of E2, the acute action of E2, which recruits non-genomic cellular mechanisms, increases risk taking in OVX females.

**References**

1. Crocker AD, Russell RW. The Up-and-Down Method for the Determination of Nociceptive Thresholds in Rats. Pharmacol Biochem Behav 1984;21:133–6.

2. Wheeler AR, Truckenbrod LM, Cooper EM, Betzhold SM, Setlow B, Orsini CA. Effects of fentanyl self-administration on risk-taking behavior in male rats. Psychopharmacology (Berl) 2023;240:2529–44.

3. Orsini CA, Blaes SL, Hernandez CM, Betzhold SM, Perera H, Wheeler AR, et al. Regulation of risky decision making by gonadal hormones in males and females. Neuropsychopharmacology 2021;46:603–13.

4. Goldman JM, Murr AS, Cooper RL. The rodent estrous cycle: characterization of vaginal cytology and its utility in toxicological studies. Birth Defects Res B Dev Reprod Toxicol 2007;80:84–97.

5. Cora MC, Kooistra L, Travlos G. Vaginal Cytology of the Laboratory Rat and Mouse: Review and Criteria for the Staging of the Estrous Cycle Using Stained Vaginal Smears. Toxicol Pathol 2015;43:776–93.

6. Marcondes FK, Bianchi FJ, Tanno AP. Determination of the estrous cycle phases of rats: some helpful considerations. Braz J Biol 2002;62:609–14.

7. Montes GS, Luque EH. Effects of ovarian steroids on vaginal smears in the rat. Acta Anat (Basel) 1988;133:192–9.

8. Orsini CA, Truckenbrod LM, Wheeler AR. Regulation of sex differences in risk-based decision making by gonadal hormones: Insights from rodent models. Behav Processes 2022;200:104663.

9. Takeo Y. Influence of continuous illumination on estrous cycle of rats: time course of changes in levels of gonadotropins and ovarian steroids until occurrence of persistent estrus. Neuroendocrinology 1984;39:97–104.

10. Orsini CA, Hernandez CM, Singhal S, Kelly KB, Frazier CJ, Bizon JL, et al. Optogenetic Inhibition Reveals Distinct Roles for Basolateral Amygdala Activity at Discrete Time Points during Risky Decision Making. J Neurosci 2017;37:11537–48.

11. St Onge JR, Abhari H, Floresco SB. Dissociable contributions by prefrontal D1 and D2 receptors to risk-based decision making. J Neurosci 2011;31:8625–33.

12. Truckenbrod LM, Cooper EM, Orsini CA. Cognitive mechanisms underlying decision making involving risk of explicit punishment in male and female rats. Cogn Affect Behav Neurosci 2023;23:248–75.

13. Frasor J, Barnett DH, Danes JM, Hess R, Parlow AF, Katzenellenbogen BS. Response-specific and ligand dose-dependent modulation of estrogen receptor (ER) alpha activity by ERbeta in the uterus. Endocrinology 2003;144:3159–66.

14. Orsini CA, Willis ML, Gilbert RJ, Bizon JL, Setlow B. Sex differences in a rat model of risky decision making. Behav Neurosci 2016;130:50–61.

15. Gonzales C, Cárdenas-Valencia I, Leiva-Revilla J, Anza-Ramirez C, Rubio J, Gonzales GF. Effects of different varieties of Maca (Lepidium meyenii) on bone structure in ovariectomized rats. Forsch Komplementmed 2010;17:137–43.

16. Mann SN, Pitel KS, Nelson-Holte MH, Iwaniec UT, Turner RT, Sathiaseelan R, et al. 17α-Estradiol prevents ovariectomy-mediated obesity and bone loss. Exp Gerontol 2020;142:111113.

17. Steinberg RM, Walker DM, Juenger TE, Woller MJ, Gore AC. Effects of perinatal polychlorinated biphenyls on adult female rat reproduction: development, reproductive physiology, and second generational effects. Biol Reprod 2008;78:1091–101.

18. Medlock KL, Forrester TM, Sheehan DM. Short-term effects of physiological and pharmacological doses of estradiol on estrogen receptor and uterine growth. J Recept Res 1991;11:743–56.

19. Kennedy TG, Armstrong DT. Loss of uterine luminal fluid in the rat: relative importance of changing peripheral levels of estrogen and progesterone. Endocrinology 1975;97:1379–85.

20. Axelson JF, Gerall AA, Albers HE. Effect of progesterone on the estrous activity cycle of the rat. Physiol Behav 1981;26:631–5.

21. Andrade S, Silveira SL, Arbo BD, Batista B a. M, Gomez R, Barros HMT, et al. Sex-dependent antidepressant effects of lower doses of progesterone in rats. Physiol Behav 2010;99:687–90.

22. Swalve N, Smethells JR, Carroll ME. Progesterone attenuates impulsive action in a Go/No-Go task for sucrose pellets in female and male rats. Horm Behav 2016;85:43–7.

23. Smethells JR, Swalve NL, Eberly LE, Carroll ME. Sex differences in the reduction of impulsive choice (delay discounting) for cocaine in rats with atomoxetine and progesterone. Psychopharmacology (Berl) 2016;233:2999–3008.

24. Gore AC, Roberts JL. Regulation of gonadotropin-releasing hormone gene expression in the rat during the luteinizing hormone surge. Endocrinology 1995;136:889–96.

25. Maffucci JA, Noel ML, Gillette R, Wu D, Gore AC. Age- and hormone-regulation of N-methyl-D-aspartate receptor subunit NR2b in the anteroventral periventricular nucleus of the female rat: implications for reproductive senescence. J Neuroendocrinol 2009;21:506–17.

**Supplemental Tables**

**Supplemental Table 1**. Mean (± standard error of the mean) locomotor activity for the Risky Decision-making Task (RDT) in Experiments 1 and 2.

|  | **Locomotor activity**  ***(locomotor units/ITI)*** | **Shock reactivity**  ***(locomotor units/shock)*** |
| --- | --- | --- |
| **Experiment 1** | |  |
| *Treatment Group* | |  |
| ***Vehicle*** | 19.21 (±2.24) | 2.84 (±0.35) |
| ***EB*** | 12.07 (±1.79)* | 2.71 (±0.47) |
|  |  |  |
| **Experiment 2** | |  |
| *Treatment Group* | |  |
| ***Vehicle*** | 14.09 (±1.71) | 2.21 (±0.25) |
| ***PPT*** | 14.68 (±1.51) | 2.19 (±0.23) |
| ***DPN*** | 14.57 (±1.79) | 2.37 (±0.30) |
| ***PPT+DPN*** | 14.45 (±1.58) | 2.08 (±0.27) |
|  |  |  |
|  |  |  |

**Indicates main effect of treatment group*

**Supplemental Figures**

**Figure S1: Effects of estradiol benzoate (EB) on ancillary behavioral measures, estrous cycle and endocrine measures. A.** Following ovariectomies (OVX), there was a significant increase in choice of the large, risky reward. **B.** Mean choice of the large, risky reward (averaged across blocks 2 through 5) over 7 days of treatment. Injections occurred after daily test sessions. Treatment with EB decreased mean choice of the large, risky reward beginning on day 3. Light blue shading indicates the period of treatment, which began after testing on day 1 and ended after behavioral testing on day 7. **C.** Treatment with EB increased latencies to press the small, safe lever during forced choice trials. **D.** Treatment with EB increased latencies to press the large, risky lever during forced choice trials. **E.** Treatment with EB significantly increased the percentage of omissions during free choice trials. **F.** In non-cycling ovariectomized (OVX) females, treatment with EB increased the number of days spent in proestrus/estrus (P/E) and decreased the number of days spent in metestrus/diestrus (M/D). **G.** Treatment with EB significantly increased serum concentrations of estradiol (E2) but did not alter serum concentrations of progesterone (P4). **H.** Treatment with EB significantly increased uterine horn width in OVX females. Data are represented as the mean of the dependent variable ± the standard error of the mean (SEM). Individual data points for each rat are displayed on bar graphs. Asterisks indicate *p*<0.05. Error bars are not displayed (e.g., 0% block in Figure S1A) when the SEM is smaller than the data point symbol.

**Figure S2. Effects of estrogen receptor (ER) agonists on ancillary behavioral measures, estrous cycle and endocrine measures. A.** Following ovariectomies (OVX), there was a significant increase in choice of the large, risky reward. **B.** Mean choice of the large, risky reward (averaged across blocks 2 through 5) over 7 days of ER agonist treatment. Injections occurred after daily test sessions. Treatment with PPT, alone or with DPN, decreased mean choice of the large, risky reward beginning on day 2. Light blue shading indicates the period of treatment, which began after testing on day 1 and ended after behavioral testing on day 7. **C.** Treatment with ER agonists, either alone or in combination, had no effect on latencies to press the small, safe lever during forced choice trials. **D.** Treatment with ER agonists, either alone or in combination, had no effect on latencies to press the large, risky lever during forced choice trials. **E.** Administration of the ERα agonist PPT, either alone or with the ERβ agonist DPN, significantly increased the percentage of omissions during free choice trials. **F.** In non-cycling ovariectomized (OVX) females, administration of PPT, either alone or with DPN, increased the number of days spent in the cytological proestrus/estrus (P/E) state and decreased the number of days spent in the cytological metestrus/diestrus (M/D) state. **G.** Treatment with ER agonists did not change serum concentrations of estradiol (E2) or progesterone (P4) in OVX females. **H.** Administration of PPT, either alone or with DPN, significantly increased uterine horn width in OVX females. Data are represented as the mean of the dependent variable ± the standard error of the mean (SEM). Individual data points for each rat are displayed on bar graphs. Asterisks indicate *p*<0.05. Error bars are not displayed (e.g., 0% block in Figure S2A) when the SEM is smaller than the data point symbol.

**Figure S3. Effects of progesterone (P4) on ancillary behavioral measures and estrous cycle. A.** Following ovariectomies (OVX), there was a significant increase in choice of the large, risky reward. **B.** Mean choice of the large, risky reward (averaged across blocks 2 through 5) over 7 days of P4 treatment, either alone or with estradiol benzoate (EB). Injections occurred after daily test sessions. Treatment with EB+P4 decreased mean choice of the large, risky reward beginning on day 3. P4 administration alone did not affect choice of the large, reward at any point during treatment. Light blue shading indicates the period of treatment, which began after testing on day 1 and ended after behavioral testing on day 7. **C.** Treatment with P4, either alone or with EB, did not affect latencies to press the small, safe lever during forced choice trials. **D.** Treatment with P4, either alone or with EB, did not affect latencies to press the large, risky lever during forced choice trials. **E.** Co-administration of EB and P4 significantly increased the percentage of omissions during free choice trials. **F.** In non-cycling ovariectomized females, co-administration of EB and P4 increased the number of days spent in proestrus/estrus (P/E) and decreased the number of days spent in metestrus/diestrus (M/D). Data are represented as the mean of the dependent variable ± the standard error of the mean (SEM). Individual data points for each rat are displayed on bar graphs. Asterisks indicate *p*<0.05. Error bars are not displayed (e.g., 0% block in Figure S3A) when the SEM is smaller than the data point symbol.

**Figure S4. Validation of progesterone (P4) doses on endocrine measures. A.** Administration of the 0.6 mg/mL dose of P4 increased serum concentrations of P4 in ovariectomized (OVX) female rats. **B.** Neither dose of P4 altered serum concentrations of estradiol (E2) in OVX females. **C.** Neither dose of P4 altered uterine horn width in OVX females. Data are represented as the mean of the dependent variable ± the standard error of the mean (SEM). Individual data points for each rat are displayed on bar graphs. Asterisks indicate *p*<0.05.

**Figure S5. Effects of acute administration of estradiol benzoate (EB) on risk taking. A.** Acute administration of EB 30 min before testing in the RDT significantly increased choice of the large, risky reward (e.g., risk taking). **B.** Acute EB administration significantly decreased choice of the small, safe reward. **C.** Acute EB administration increased the percentage of win-stay trials but had no effect on the percentage of lose-shift trials. **D.** Acute EB administration did not alter latencies to press the small, safe lever during forced choice trials. **E**. Acute EB administration did not alter latencies to press the large, risky lever during forced choice trials. **F.** Acute EB administration significantly decreased the percentage of omissions during free choice trials. Data are represented as the mean of the dependent variable ± the standard error of the mean (SEM). Individual data points for each rat are displayed on bar graphs. Asterisks indicate *p*<0.05. Error bars are not displayed (e.g., 0% block in Figure S5A) when the SEM is smaller than the data point symbol.

**Figure S1**


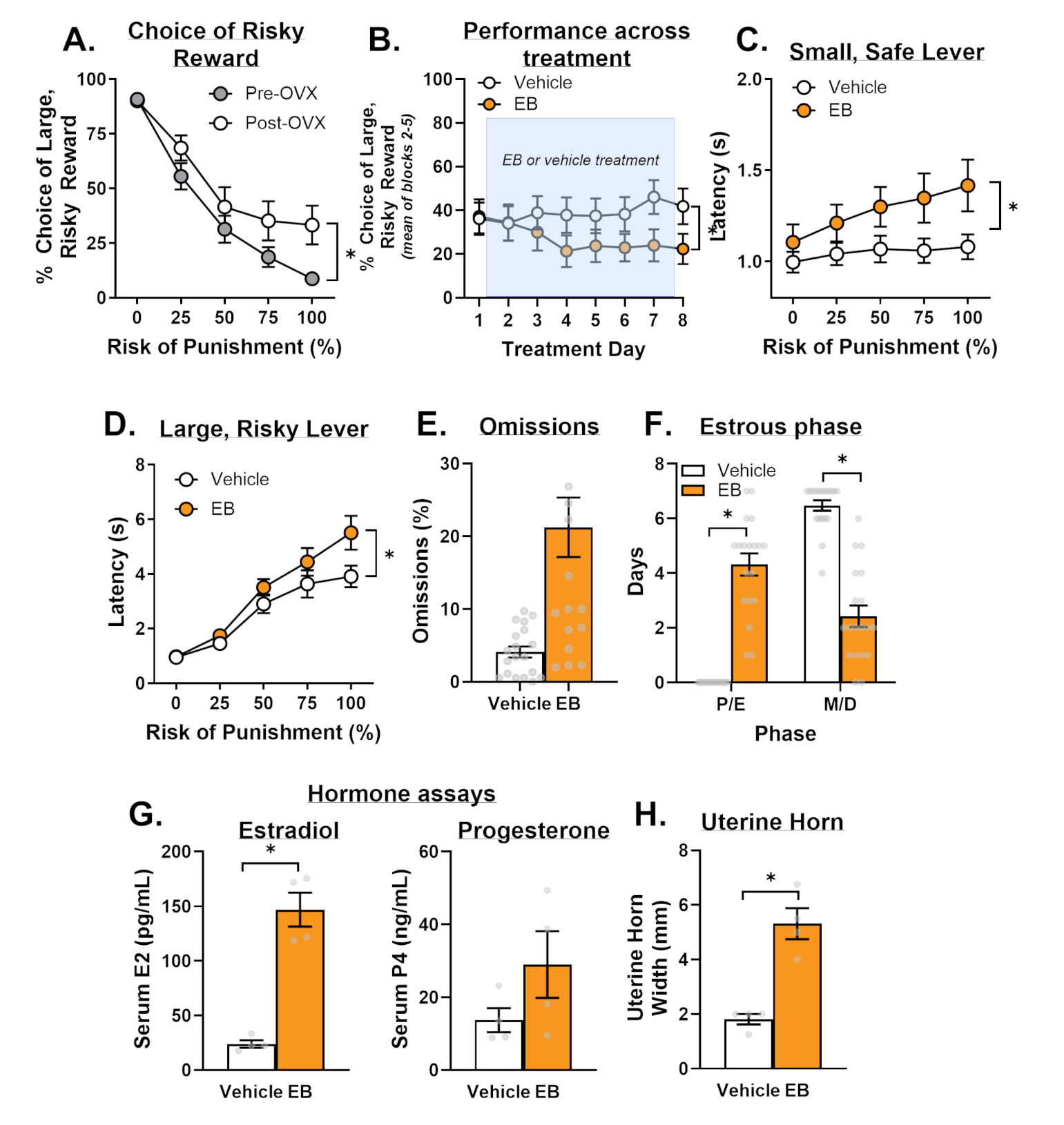


**Figure S2**


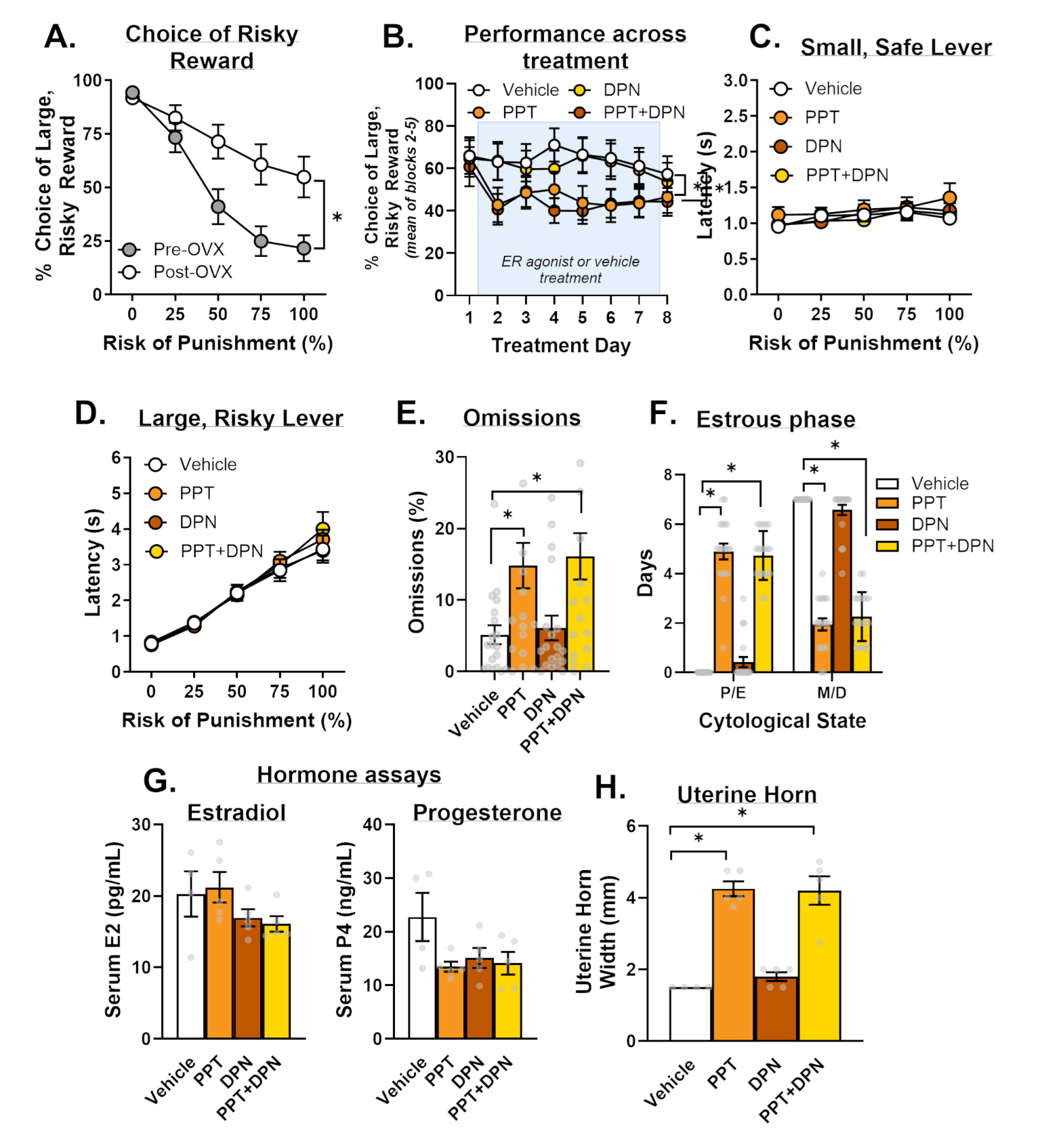


**Figure S3**


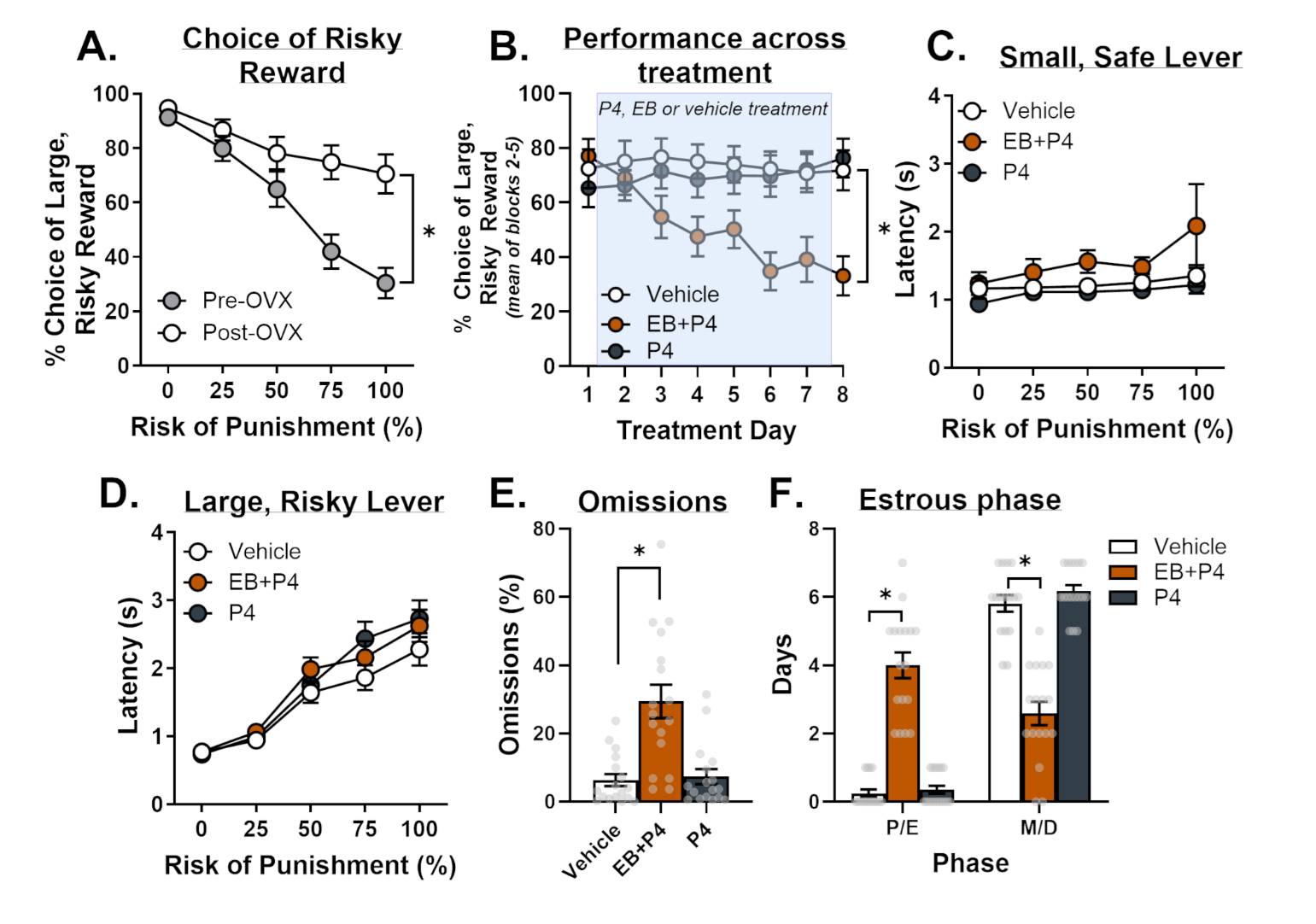


**Figure S4**


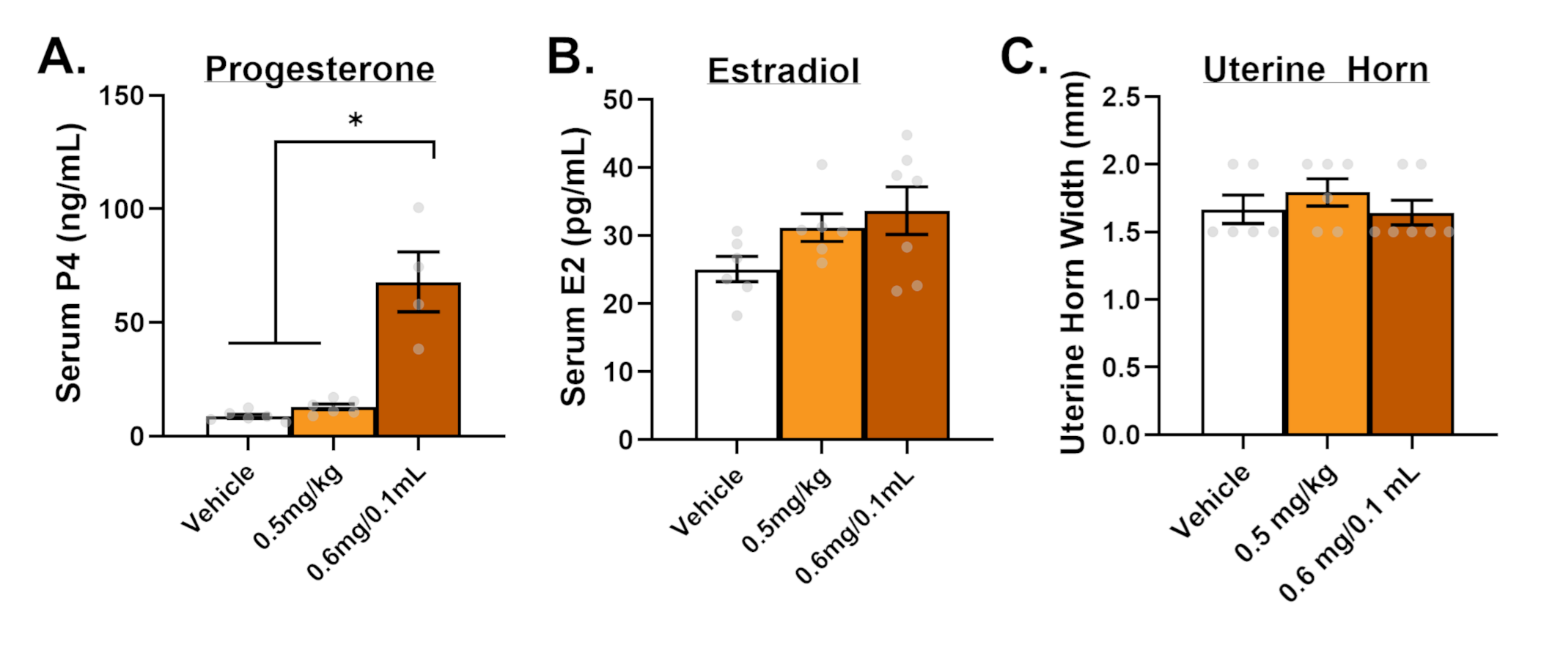


**Figure S5**


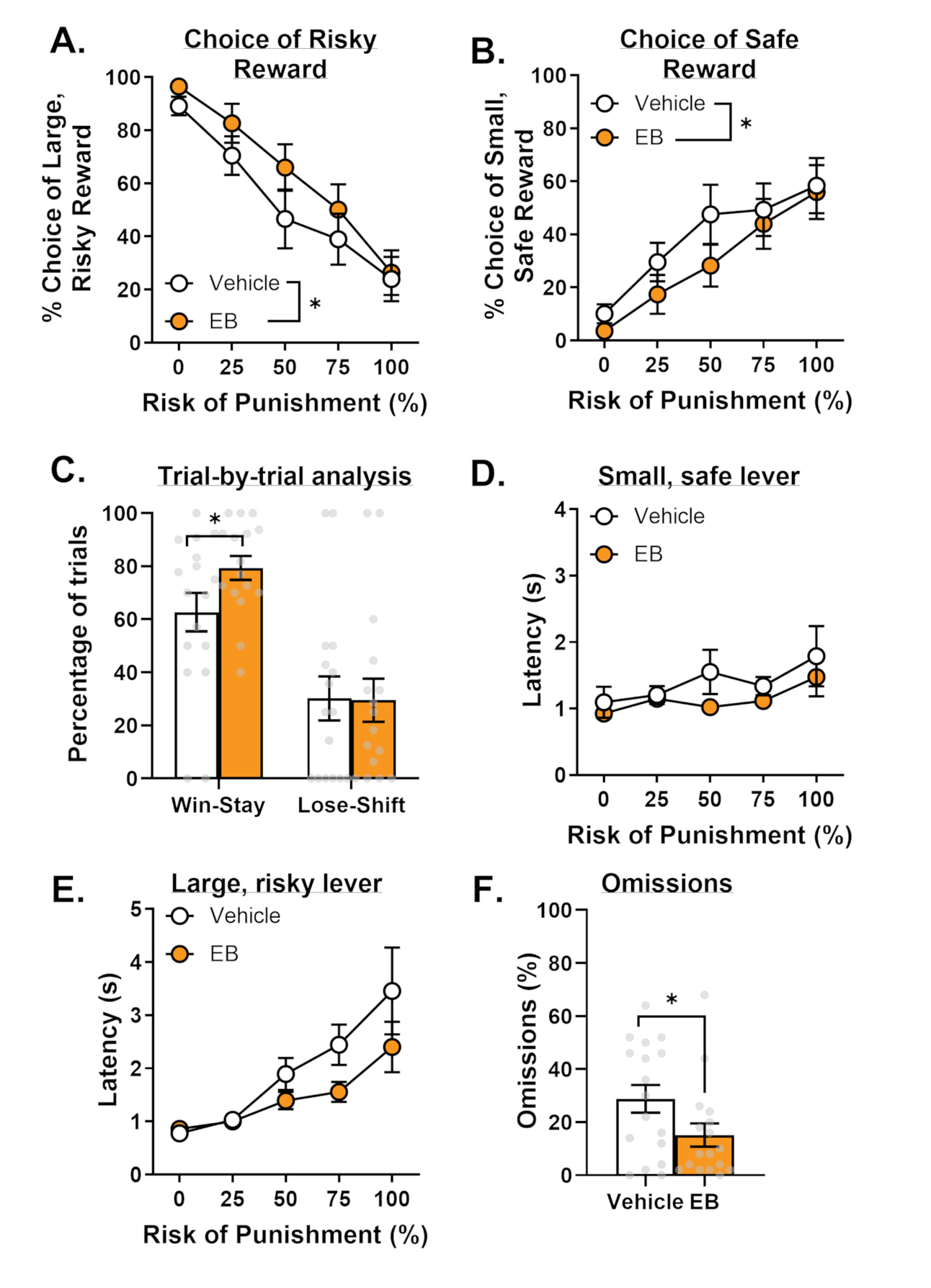

Supplement: Supplementary file 1 — Supplementary Material [file 41386_2026_2347_MOESM1_ESM.docx]
